# Personalised virtual gene panels reduce interpretation workload and maintain diagnostic rates of proband-only clinical exome sequencing for rare disorders

**DOI:** 10.1136/jmedgenet-2020-107303

**Published:** 2021-04-20

**Authors:** Leslie Patricia Molina-Ramírez, Claire Kyle, Jamie M Ellingford, Ronnie Wright, Algy Taylor, Sanjeev S Bhaskar, Christopher Campbell, Harriet Jackson, Adele Fairclough, Abigail Rousseau, George J Burghel, Laura Dutton, Siddharth Banka, Tracy A Briggs, Jill Clayton-Smith, Sofia Douzgou, Elizabeth A Jones, Helen M Kingston, Bronwyn Kerr, John Ealing, Suresh Somarathi, Kate E Chandler, Helen M Stuart, Emma MM Burkitt-Wright, William G Newman, Iain A Bruce, Graeme C Black, David Gokhale

**Affiliations:** 1 Division of Evolution and Genomic Sciences, School of Biological Sciences, Faculty of Biology, Medicine and Health, The University of Manchester, Manchester, UK; 2 North West Genomic Laboratory Hub, Manchester Centre for Genomic Medicine, Manchester University NHS Foundation Trust, Manchester, Greater Manchester, UK; 3 Manchester Centre for Genomic Medicine, Manchester University NHS Foundation Trust, Manchester, UK; 4 Department of Neurology, Salford Royal NHS Foundation Trust, Salford, Salford, UK; 5 Division of Infection, Immunity and Respiratory Medicine, Faculty of Biology, Medicine and Health, The University of Manchester, Manchester, UK; 6 Paediatric ENT Department, Royal Manchester Children's Hospital, Manchester University NHS Foundation Trust, Manchester, UK

**Keywords:** genomics, early diagnosis, genetics, medical

## Abstract

**Purpose:**

The increased adoption of genomic strategies in the clinic makes it imperative for diagnostic laboratories to improve the efficiency of variant interpretation. Clinical exome sequencing (CES) is becoming a valuable diagnostic tool, capable of meeting the diagnostic demand imposed by the vast array of different rare monogenic disorders. We have assessed a clinician-led and phenotype-based approach for virtual gene panel generation for analysis of targeted CES in patients with rare disease in a single institution.

**Methods:**

Retrospective survey of 400 consecutive cases presumed by clinicians to have rare monogenic disorders, referred on singleton basis for targeted CES. We evaluated diagnostic yield and variant workload to characterise the usefulness of a clinician-led approach for generation of virtual gene panels that can incorporate up to three different phenotype-driven gene selection methods.

**Results:**

Abnormalities of the nervous system (54.5%), including intellectual disability, head and neck (19%), skeletal system (16%), ear (15%) and eye (15%) were the most common clinical features reported in referrals. Combined phenotype-driven strategies for virtual gene panel generation were used in 57% of cases. On average, 7.3 variants (median=5) per case were retained for clinical interpretation. The overall diagnostic rate of proband-only CES using personalised phenotype-driven virtual gene panels was 24%.

**Conclusions:**

Our results show that personalised virtual gene panels are a cost-effective approach for variant analysis of CES, maintaining diagnostic yield and optimising the use of resources for clinical genomic sequencing in the clinic.

## Introduction

Exome (ES) and genome sequencing (GS) approaches are now commonplace in healthcare settings, enabling the identification and assessment of a broad spectrum of variants which may be causative of monogenic disorders. Clinical ES and GS strategies have demonstrated advantages over other diagnostic testing techniques, as they are capable of identifying previously undetected pathogenic variants, including those in genes not previously surveyed through custom gene panel or single gene approaches. Such findings can improve diagnostic yields and thereby guide appropriate patient management and therapeutic options. The diagnostic yield of clinical ES approaches is wide-ranging with reported rates between 20% and 50%,^1–9^ impacted by cohort size and clinical characteristics. ES approaches have been shown to have reduced diagnostic yield compared with custom gene panel approaches dependent on patient recruitment criteria.^10^

The speed of variant interpretation remains an important challenge in the adoption of NGS as a clinical diagnostic test. NGS gene panels and, in particular, ES and GS generate large and complex volumes of data. The number of potentially pathogenic variants identified through ES and GS place a considerable burden on accredited medical genomic services in analysing and interpreting variants within a clinical setting, which may reduce accuracy and efficiency. The workload of interpretation is an important consideration when implementing genomic sequencing in the clinic. Cost-effective and accurate interpretation of genetic variants is fundamental to the widespread implementation of ES and GS in clinical settings, but currently, techniques to address this challenge in clinical contexts has been poorly assessed in current diagnostic practice.

In this study, we examined the use of a clinician-led and phenotype-driven approach to semi-automatic generation of personalised virtual gene panels. We evaluated the impact of this approach on diagnostic yield and interpretative workload, in the context of diagnosis of patients with presumed rare monogenic disease in an accredited clinical genomic medicine service.

## Methods

### Cohort description and virtual gene panel generation

We conducted a retrospective survey of clinical exome variant results from patients with rare disease undergoing CES from October 2016 to January 2020. All CES data analyses and results described in this study were undertaken in a UK NHS Accreditation Service Clinical Pathology Accredited Medical Laboratory. Patients or guardians specifically consented for CES data analysis and data sharing as part of the diagnostic investigations and for results audit purposes. Written informed consent was obtained from patients or guardians explaining benefits and risks of CES testing.

Patients were referred by consultant clinical geneticists using a web-based referral system (WRS) to capture patient demographics, clinical features and/or Human Phenotype Ontology (HPO) terms to facilitate semi-automated generation of personalised virtual gene panels. Information entered into the WRS was used to create virtual gene panels using one or more of the following methods:

- Curated gene-disease panels: selection of curated gene lists from Genomics England PanelApp^11 12^ and/or previous UK Genetic Testing Network panels.^13^
- HPO-based gene selection: selection of HPO terms^14^ generates a list of candidate genes from OMIM^15^ and Orphanet.^16^
- Customised selection of genes specified by clinicians, based on their clinical hypotheses.

HPO-based gene lists are created automatically on the WRS through a custom algorithm developed to use HPO terms intelligently, allowing additional terms entered to increase specificity of the panel rather than simply increasing in size (online supplemental methods figure S2). The inclusion of genes that are present on the American College of Medical Genetics and Genomics (ACMG) incidental findings list^17^ was performed at the discretion of the clinician, with prior consent from the patient accordingly. Genes with low predicted coverage were flagged to the referrer during the submission process.

10.1136/jmedgenet-2020-107303.supp1 Supplementary data

### Targeted clinical exome sequencing

#### Sequencing, read mapping and variant calling

DNA was isolated from peripheral blood samples (n=393) and umbilical cord samples (n=7) and CES conducted using a custom-designed Agilent SureSelect XT Focused Exome capture library and the Illumina NextSeq 500 sequencing system with 75 bp paired end reads. Sequencing was conducted to a mean depth of 112 with >97% bases covered at 20× read depth. Reads generated by the Illumina NextSeq were aligned with BWA-MEM (V.0.6.2) to the human genome build GRCh37(hg19), with local realignment performed by ABRA (V.0.96). Variant calling was subsequently carried out using SamTools (V.0.1.18/gcc-4.4.6) for SNPs and small indels and Pindel (V.0.2.4.t) for indels >5 bp.

#### Variant prioritisation and pathogenicity evaluation

Sample-specific genome alignment (.BAM) and variant (.VCF) files were analysed using Golden Helix VarSeq software (V.1.4.4).^18^ The VarSeq software interface allows users to customise configurable workflows for variant prioritisation. Annotation of variants was performed according to RefSeq: NCBI RefSeq Annotation Release 105^19^ using the most clinically relevant GRCh37(hg19) transcript. The selection of the clinically relevant transcript by VarSeq is typically based on ACMG guidelines and ClinVar’s algorithm for transcript selection. Only variants present within coding exons and +10 bp of the splice site junction were retained for analysis. Missense variants were analysed using a number of in silico predictors (eg, dbNSFP Function Predictions 3.0,^20^ GHI (SIFT),^21^ PolyPhen-2,^22^ MutationTaster Mutation Assessor, FATHMM). Putative splicing variants were analysed using Alamut V.2.4.5, dbscSNV Splice Altering Predictions 1.1, GHI and SPIDEX.^23^ Rare, high-quality, high-impact (genotype quality >20, read depth >10) single nucleotide variants and small indels with alternative allele frequency (AAF) <0.001 (gnomAD) were filtered. Variants listed as pathogenic or likely pathogenic on NCBI ClinVar were retained if present at 2% AAF. An example of our variant filtering is illustrated in online supplemental methods figure S1.

Filtered variants were analysed independently by two registered clinical scientists and classified according to ACMG guidelines^24^ into one of five classes: (i) benign, (ii) likely benign, (iii) uncertain significance, (iv) likely pathogenic and (v) pathogenic. Variants were validated through Sanger sequencing. Segregation studies in parents of patients with possible compound heterozygous variants performed. Where needed, cases are reviewed at internal multidisciplinary team meetings or through internal communication between the clinical scientist and the clinical geneticist.

### Retrospective evaluation of clinical characteristics and diagnostic rate of CES results

Clinical characteristics of the cohort were determined using the information available in their referrals (HPO terms, clinical descriptions). We used the phenotypic abnormality subontology of the HPO to classify the clinical characteristics of the cohort.

To determine the diagnostic rate, CES results were categorised as described in box 1. We analysed the diagnostic rate reported in relation to the main clinical referral indications for referral and the methods used for virtual gene panel generation.

Box 1 **Categorisation of CEs diagnosis**

**Diagnosis confirmed**

In a clinically relevant gene, the presence of either:

1. A heterozygous class 4 or 5 variant in a dominant condition,
2. A homozygous/hemizygous class 4 or 5 variant in a recessive condition or
3. Two class 4 or 5 variants in the same gene in a recessive condition (potential compound heterozygote).

**Diagnosis possibly confirmed**

In a clinically relevant gene, the presence of either:

1. A homozygous/hemizygous class 3 variant* in a recessive condition,
2. A class 3 variant* and a class 4 or 5 variant in the same gene in a recessive condition (potential compound heterozygote) or
3. A heterozygous class 3 variant* in a dominant condition where parental studies suggest a possible de novo.

**Diagnosis not confirmed**

In a clinically relevant gene, the presence of either:

1. Any heterozygous class 3, 4 or 5 variant in a recessive condition,
2. A heterozygous class 3 variant* in a dominant condition where further parental testing has not been performed or
3. No plausibly causative variant identified.

### Quantification of variant workload

We quantified the number of variants prioritised per sample. We then removed the virtual gene panel file and determined the number of variants obtained without the virtual gene panel and prioritising loss-of-function (LOF) and missense PP3 classified-variants (ACMG-AMP system^24^). A comparison was made between variant workloads with and without use of virtual gene panels in order to determine the impact of using virtual gene panels in the clinical scientist’s interpretative workload.

### Statistical methods

Descriptive statistics (mean, median and SD) were used for phenotypic descriptions, virtual gene panel size and variant workload. One-sample binomial test was used to determine gender differences, χ^2^ test was used for categorical variables where applicable, Spearman’s rank correlation was used to determine the relationship between variant workload and total number of genes included in virtual gene panels. Statistical significance was denoted as p<0.05. R V.3.5 and IBM SPSS V.23 were used for analysis.

## Results

### Clinical characteristics of referrals

A total of 400 patients with presumed rare disorders were referred as singletons from October 2016 to December 2019 for diagnostic targeted CES. A total of 273 cases were under age 18 years at the time of referral (69%, 273/394). Of these, 60% (164/273) were aged 5 years or less. Six referrals came from fetal samples. No significant difference in gender ratio was found (46.7% female vs 53.3% male; p=0.208, one sample binomial test).

Referred cases presented with a wide range of phenotypic characteristics. No significant difference was observed between the number of cases referred with one organ or system affected (50.5%, 202/400) in comparison to those presenting phenotypic abnormalities affecting two or more organs (49.5%, 198/400). Phenotypic abnormalities of the nervous system were present in more than half of the referrals (54.5%, n=218/400) (online supplemental figure 1). Of these, intellectual disability and/or developmental delay was present in 43.1% (94/218). Other major phenotypic abnormality categories were described by terms denoting conditions in head and neck (19%, 74/400), the skeletal system (16%, 65/400), ear (15%, 59/400), eye (15%, 58/400), and growth abnormalities (13%, 51/400).

10.1136/jmedgenet-2020-107303.supp2 Supplementary data

### Virtual gene panel generation

In more than half of the patients (57%, 226/400), virtual gene panels were generated by the clinical referrer combining gene selection methods. The use of curated panels (eg, PanelApp), either as a single method or in combination with one or more method(s), was the most common approach (73%, 291/400) followed by HPO-based gene lists (56%, 223/400) (online supplemental figure 2A). The combined selection of HPO-based gene lists and curated panels was mostly used in cases with two or more organs or systems affected. However, no significant difference was observed in the utilisation of a specific virtual gene panel method for the main clinical indications (ie, abnormalities in nervous system, ear, eye, head or neck, skeletal system and musculature abnormalities; χ^2^ p=0.28,online supplemental figure 2B).

Virtual gene panels included a median of 107 genes (mean 233, range 1–1380, SD 296, 95% CI 204.30 to 262.49). Combining gene selection approaches generated larger virtual gene panels (n=226, median=165.5 genes) than those generated by one approach (n=174, median=64 genes, online supplemental figure 3A) (p<0.05, one sample Wilcoxon signed-rank test). When examining single methods, curated panels contained more genes in comparison to single HPO-based selection (p=0.0011, Wilcoxon signed-rank test). Virtual gene panels were significantly larger for cases with presence of more than two organs or systems affected (n=198, median 142 genes) in comparison to those with phenotypic abnormalities affecting only one single organ/system (p<0.05, Wilcoxon signed-rank test).

### Impact of virtual gene panels on reduction of variant workload

**Figure 1:**
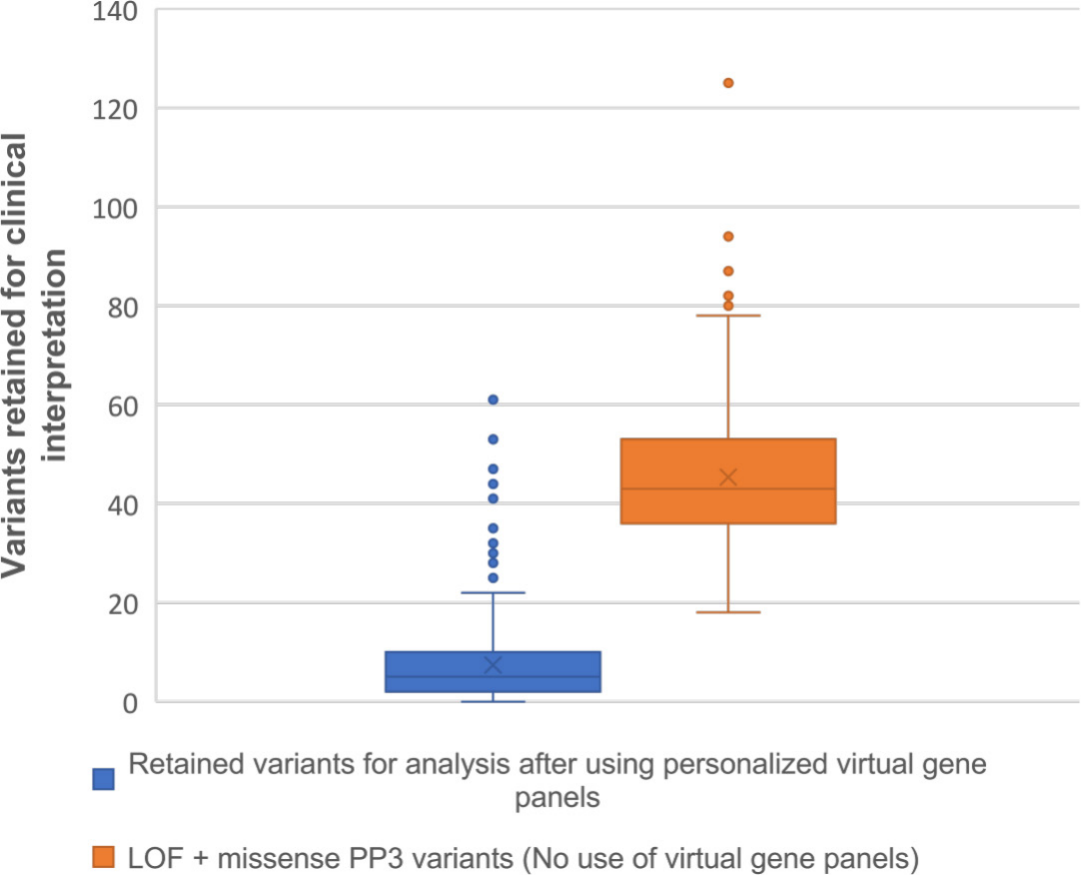
Comparison of variants retained for clinical interpretation between the use of personalised virtual gene panels and prioritisation of loss-of-function (LOF) and missense PP3 variants 20 without phenotype-based virtual gene panels.

The average number of filtered variants for interpretation per sample was 7.38 (median=5 variants), ranging from 0 to 61 variants (95% CI 6.58 to 8.18, online supplemental figure 3B). A significant correlation was observed between panel size and variant workload (r^2^=0.76, p<0.05 Spearman’s rank, online supplemental figure 4). We sought to compare the reported variant workload after using virtual gene panels with the variant volume produced by filtering LOF and missense with 4/6 in silico evidence of pathogenicity without phenotype-based targets. The latter prioritisation method led to an average of 45.3 variants, within a range between 18 and 125 (95% CI 44.01 to 46.68), showing a significant difference when compared with the variant workload obtained by using virtual gene panels (p<0.0001, unpaired t-test, figure 1).

### Molecular results and diagnostic rate

A total of 180 variants across the 400 clinical exomes were identified and assigned ACMG classification scores^24^ (online supplemental table 1). Pathogenic and likely pathogenic variants accounted for 62.2% of these (112/180). Sixty per cent were LOF (67/112) and 40% were missense (45/112). Only one variant was reported synonymous. Novel likely pathogenic and/or pathogenic variants at the time of original analysis accounted for 32.1% (36/112). Variants of uncertain significance accounted for 37.2% (68/180).

10.1136/jmedgenet-2020-107303.supp3 Supplementary data

Overall, 24% of the patients received a confirmed and/or possibly confirmed molecular diagnosis (96/400). Seven additional cases (1.75%) had a genetic finding reported that confirmed only part of the phenotype—in one case with suspected digenic inheritance. The inclusion of these cases would increase the diagnostic rate to 26.75%. Findings associated with autosomal recessive inheritance were reported in 59.3% (58/96), autosomal dominant in 30% (32/96), X linked dominant in 5.2% (5/96) and X linked recessive inheritance in 1% (1/96). In two cases, likely pathogenic variants were identified in genes that have been associated with autosomal dominant and autosomal recessive inheritance. Single heterozygous variants in genes associated with autosomal recessive inheritance were identified in 2.5% of the cohort (10/400); these were reported in cases with presumed autosomal recessive conditions.

Variants of unknown significance identified in clinically relevant genes were considered for diagnosis in specific cases. Ten possibly confirmed cases were reported with a variant of unknown significance (VUS) in potential compound heterozygous state with a likely pathogenic or pathogenic variant. Other nine possibly confirmed cases were reported with homozygous variants for disorders with autosomal recessive inheritance. Four cases were reported with confirmed de novo VUS in patients with autosomal dominant disorders. Cases with a reported heterozygous class 3 in an autosomal dominant condition where further parental testing has not been performed were not considered as plausible confirmation of diagnosis. No incidental findings were identified or reported, as expected by the gene selection approach.

The diagnostic rate for the most common phenotypic categories ranged between 21.5% and 32.7% (online supplemental figure 1B). No differences were observed in diagnostic yield when comparing the rate across the different methods for virtual gene panel generation (p=0.347) (online supplemental figure 5).

## Discussion

Identifying approaches to efficiently sift variants from ES and GS analyses for clinical interpretation can have considerable benefit in rationalising workstream flows in clinical diagnostic laboratories. Phenotype-driven approaches have become widely available tools for variant^25–27^ and gene prioritisation^28 29^ of genomic sequencing data. Therefore, incorporating detailed clinical phenotyping alongside CES and CGS offers an opportunity to develop personalised testing strategies for patients with rare disease through virtual gene panels.

Evidence has shown that CES using virtual gene panels can be an effective option for investigating individuals with rare Mendelian monogenic disorders.^29 30^ In this study, we show that the availability of different phenotype-based approaches to gene selection can be beneficial for the design of personalised virtual gene panels. This is consistent with the increased sensitivity reported by Maver *et al* using phenotype-based virtual gene panels.^29^ Notably, each virtual gene panel method is characterised by a set of features that can complement one another. Curated disease-gene panels (eg, PanelApp) are comprehensive, expertly curated evidence-based lists of genes. However, depending on the condition, they may contain genes that are irrelevant to the specific patient case. In this case, the sole or concomitant use of HPO-based gene selection may produce a more personalised selection that can be further improved as gene-disease associations increase over time.^31 32^ Cases with atypical or unclear clinical diagnoses may certainly benefit from combined approaches. Similarly, clinical acumen can add sensitivity to panel design. Offering gene selection options based on different methods or algorithms facilitates the clinicians’ choice of the most adequate approach for their patients and, if necessary, allows the combination of methods to increase the probability of inclusion of relevant genes in the panel.

We also show that the use of personalised virtual gene panels can increase the efficiency of clinical variant analysis strategies without compromising diagnostic yield. Our result is consistent with diagnostic rates reported to date for CES in clinical settings.^2 4 33 34^ The diagnostic rates observed in the main five phenotypic categories (online supplemental figure 1) also highlight the utility of the singleton CES approach in the investigation of a breadth of frequent clinical indications for CES.^5 35^ Furthermore, we expect that our diagnostic yield to be further improved following the introduction of parallel CNV analysis.^36^ This addition would be particularly useful in cases where a heterozygous variant was detected in a phenotypically relevant recessive gene (3%, 10/400), where a second variant in trans is suspected beyond the detection of our current approach may be suspected.

Understanding the efficiency of variant analysis strategies is of paramount importance in clinical laboratories. A number of variant filtering and/or ranking strategies are available.^25 26 37^ Here, we show that variant analysis within a personalised gene selection is both a sensitive and more efficient technique to apply to CES datasets. This is evidenced by the reduced variant workload produced by using personalised virtual gene panels (median=5 variants) in comparison to the total variants retained after using the same filtering algorithm without virtual gene panels and prioritising LOF and missense variants with ACMG-criterion PP3 (median=43 variants). Our reported number of filtered variants per case (mean=7.38, median=5) is noticeably lower than the average reported in some studies that use phenotype-driven gene selection methods for ES analysis. Kernohan *et al* identified a minimum average variant burden ranging from 42 to 46 variants using Radboudumc and HPO-based panels in singleton-ES cases, respectively.^38^ Bergant *et al* reported a total of 91 coding variants per case after initial ES analysis.^39^ When searching for characteristics of our variant prioritisation workflow that could influence variant burden, we found that retaining very low frequency variants that occur in <0.1% of the population is an additional important factor for reduction of variant workload. Our findings suggest that the flexibility to choose the most adequate gene selection approach for virtual gene panel generation, in addition to filtering very low frequency variants, is an effective strategy that offers a deliverable variant analysis burden and maintains diagnostic efficacy in the clinical setting. Furthermore, more than half of the variants detected in our study were LOF and/or previously reported variants at the time of original analysis. It is possible to suggest that automated prioritisation of these variants could further expedite the variant sifting process. Future work incorporating variant zygosity and disease inheritance patterns, such as that developed by the Transforming Genetic Medicine Initiative^40^ may further increase the sensitivity and efficiency of CES methodologies.

In summary, the utilisation of personalised virtual gene panels represents a sustainable approach for targeted clinical exome sequencing in patients with rare disease. It can reduce interpretative variant workload and preserve diagnostic yield and potentially maintain a deliverable timeframe for clinical laboratories. Importantly, the use of different phenotype-based strategies for gene selection plays a key role for optimal gene selection. In addition, semi-automated prioritisation of previously reported variants in addition to LOF variants could further expedite interpretative workload. These strategies altogether can potentially free up time for the investigation of more complex cases and to increase analysis throughput. The optimisation of approaches and resources for data analysis will allow a deeper adoption of genomic strategies as routine practice for personalised medicine in the clinic.

## Data Availability

All data relevant to the study are included in the article or uploaded as supplementary information.
