# Supplementary material for: Personalised virtual gene panels reduce interpretation workload and maintain diagnostic rates of proband-only clinical exome sequencing for rare disorders
Source: J Med Genet. 2021 Apr 20;59(4):393–8. doi: 10.1136/jmedgenet-2020-107303 (PMC8961756; doi:10.1136/jmedgenet-2020-107303)
Supplement: Supplementary data [file jmedgenet-2020-107303supp001.pdf]

Personalised Virtual Gene Panels Reduce Interpretation Workload and Maintain Diagnostic Rates of Proband-Only Clinical Exome Sequencing for Rare Disorders.

Molina-Ramírez LP[1,2] , Kyle C[2], Ellingford JM[1,2], Wright R[2], Taylor A[2], Campbell C[2], Jackson H[2], Fairclough A[2] , Rousseau A[2], Burghel G[2], Dutton L[2], Banka S[1,2], Briggs TA[1,2], Clayton-Smith J[1,2], Douzgou S[1,2], Jones EA[1,2], Kingston H[2], Kerr B[2], Ealing J[2,3], Somarathi S[2], Chandler K[2], Stuart HM [1,2] , Burkitt-Wright E[1,2], Newman WG[1,2], Bruce IA[4,5], Black GC[1,2] (corresponding), Gokhale D[2].

1.-Division of Evolution and Genomic Sciences, School of Biological Sciences, Faculty of Biology, Medicines and Health, University of Manchester, Manchester Academic Health Science Centre, Manchester, M13 9PL, UK
2.-NW Genomic Laboratory Hub, Manchester Centre for Genomic Medicine, St. Mary's Hospital, Manchester University NHS Foundation Trust, Oxford Road Manchester M13 9WL, UK
3.- Department of Neurology, Salford Royal Foundation Trust, Manchester Academic Health Sciences Centre (MAHSC), Manchester, UK
4.-Paediatric ENT Department, Royal Manchester Children's Hospital, Manchester University Hospitals NHS Foundation Trust, Manchester Academic Health Science Centre.
5.-Division of Infection, Immunity and Respiratory Medicine, Faculty of Biology, Medicine and Health University of Manchester, Manchester, UK.

Supplementary methods

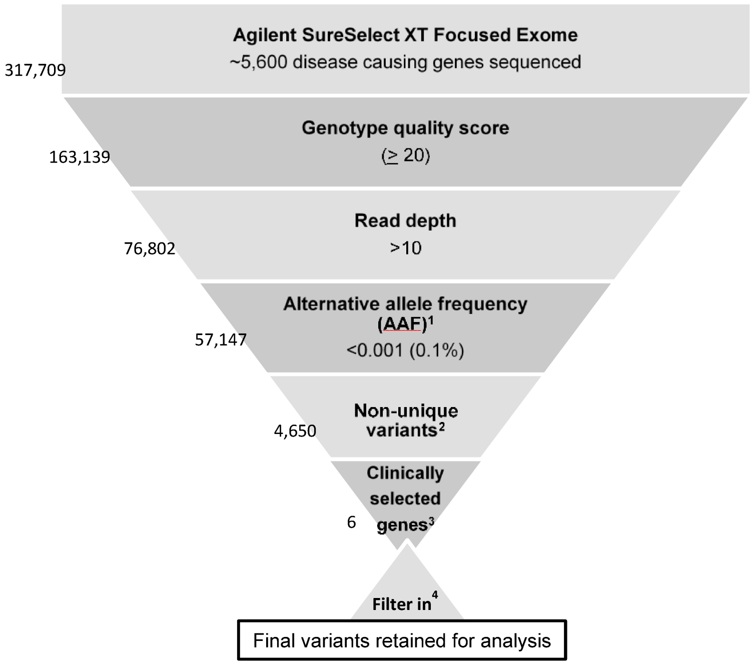

Methods figure S1. Variant filtering and prioritisation strategy. Number of variants indicated on the left represents a common example of filtering for low frequency high impact variants. Number on the left indicates example of retained variants after each filter. 1 = gnomAD. 2 = non-unique variants defined as those present in other run samples (max 12 run samples). 3 = Virtual gene panels (Coding exons +/- 10bp of splice site junction). 4 = Variants listed as “Pathogenic” or “Likely pathogenic” on NCBI ClinVar with an AAF <2% are filtered in.

. .

$$
\begin{aligned}
D = {} & \pi_{gene}, \\
& \rho c_{COUNT(phenotype)}(P), \\
& \rho p \frac{COUNT(phenotype)(P)}{COUNT(phenotype)C} \\
& \sigma_{phenotype} P \in C \\
& \gamma_{gene} P
\end{aligned}
$$

$$
\begin{aligned}
S = {} & \pi D, \\
& \rho m_{MAX(c)}(D) \\
& \sigma_{p>a} D, \\
& \tfrac{c}{m} > r
\end{aligned}
$$

$\sigma_{phenotype} P \in C$	Retrieve (σ) all genes that are linked to a phenotype (phenotypeP) that is in the list (∈) specified by the clinician (C)
$\gamma_{gene} P$	And for each (γ) gene (geneP) in the data
$D = \pi_{gene}$,	Get (π) the gene symbol (gene)
$\rho c_{(COUNT(phenotype))}$,	Get the total number of phenotypes (COUNT(phenotype)) linked to the gene, and name it c (ρc)
$\rho p_{(COUNT(phenotype))}(P) / _{COUNT(phenotype)}C$	Get the percentage of linked phenotypes (COUNT(phenotype)(P)) relative to the number specified by the clinician (COUNT(phenotype)C) and call it p (ρp)

$S = \pi D$,	Get (π) all of the data we've calculated so far (D)
$\rho m_{max(c)}(D)$	Find the gene that's been linked to the largest number of phenotypes (max(c)(D)), and record how many phenotypes it's been linked to as "m" (ρm)
$\sigma_{p>a} D$,	Filter the list of linked genes (σ), where the number of phenotypes each gene is linked to, relative to the total specified by the clinician (p) is greater than the absolute threshold (p > aD)
$(c/m)>r$	And that the number of phenotypes it's linked to relative to the best-matched gene (c / m) is more than the relative threshold (> r)

Methods figure S2. Relational algebra formulas applied to the Human Phenotype Ontology (HPO) based gene selection for generating personalised virtual gene panels
