# Supplementary material for: Personalised virtual gene panels reduce interpretation workload and maintain diagnostic rates of proband-only clinical exome sequencing for rare disorders
Source: J Med Genet. 2021 Apr 20;59(4):393–8. doi: 10.1136/jmedgenet-2020-107303 (PMC8961756; doi:10.1136/jmedgenet-2020-107303)
Supplement: Supplementary data [file jmedgenet-2020-107303supp002.pdf]

Personalised Virtual Gene Panels Reduce Interpretation Workload and Maintain Diagnostic Rates of Proband-Only Clinical Exome Sequencing for Rare Disorders.

Molina-Ramírez LP[1,2] , Kyle C[2], Ellingford JM[1,2], Wright R[2], Taylor A[2], Campbell C[2], Jackson H[2], Fairclough A[2] , Rousseau A[2], Burghel G[2], Dutton L[2], Banka S[1,2], Briggs TA[1,2], Clayton-Smith J[1,2], Douzgou S[1,2], Jones EA[1,2], Kingston H[2], Kerr B[2], Ealing J[2,3], Somarathi S[2], Chandler K[2], Stuart HM [1,2] , Burkitt-Wright E[1,2], Newman WG[1,2], Bruce IA[4,5], Black GC[1,2] (corresponding), Gokhale D[2].

1.-Division of Evolution and Genomic Sciences, School of Biological Sciences, Faculty of Biology, Medicines and Health, University of Manchester, Manchester Academic Health Science Centre, Manchester, M13 9PL, UK
2.-NW Genomic Laboratory Hub, Manchester Centre for Genomic Medicine, St. Mary's Hospital, Manchester University NHS Foundation Trust, Oxford Road Manchester M13 9WL, UK
3.- Department of Neurology, Salford Royal Foundation Trust, Manchester Academic Health Sciences Centre (MAHSC), Manchester, UK
4.-Paediatric ENT Department, Royal Manchester Children's Hospital, Manchester University Hospitals NHS Foundation Trust, Manchester Academic Health Science Centre.
5.-Division of Infection, Immunity and Respiratory Medicine, Faculty of Biology, Medicine and Health University of Manchester, Manchester, UK.

BMJ Publishing Group Limited (BMJ) disclaims all liability and responsibility arising from any reliance placed on this supplemental material which has been supplied by the author(s)

Supplementary figures

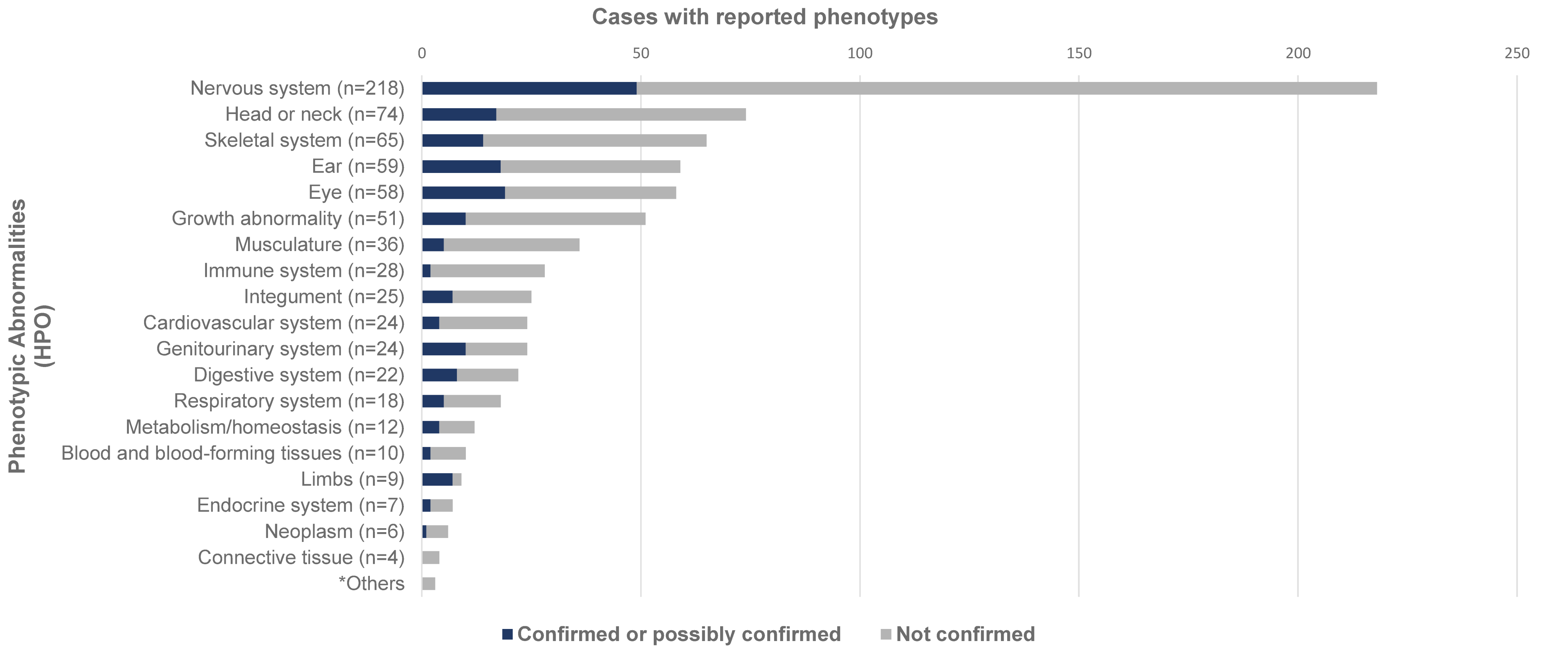

Supplementary Figure 1. Representation of phenotypic abnormalities reported in patients referred for targeted exome sequencing and proportion of cases where a molecular diagnosis was confirmed or possibly confirmed.

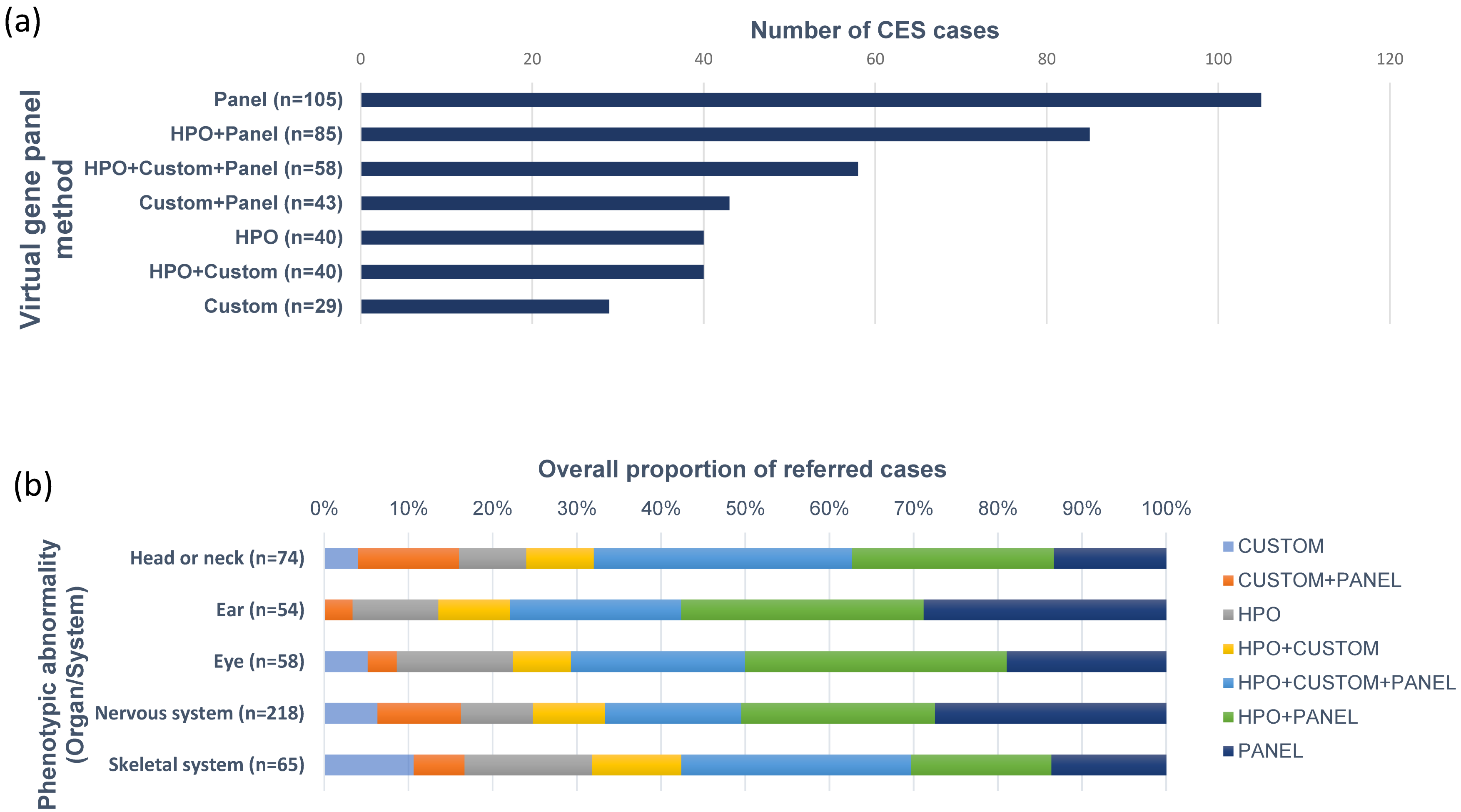

Supplementary Figure 2. (a) Total of cases per method(s) used for virtual gene panel generation. (b) Utilisation of method(s) for virtual gene panel generation across the main phenotypic categories.

(a) (b)

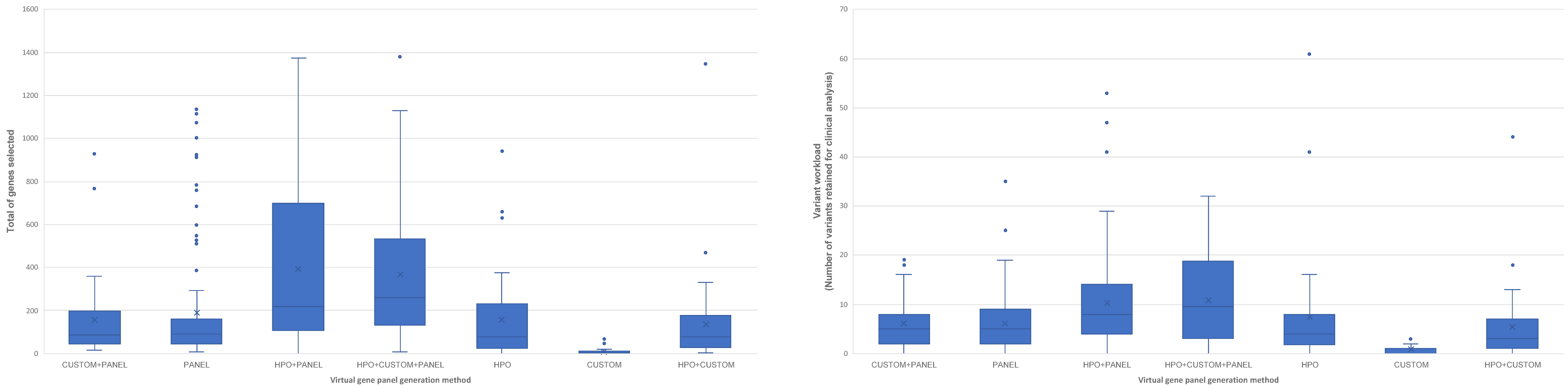

Supplementary Figure 3. (a) Comparison of panel size and (b) variant workload across the different methods for virtual gene panel generation.

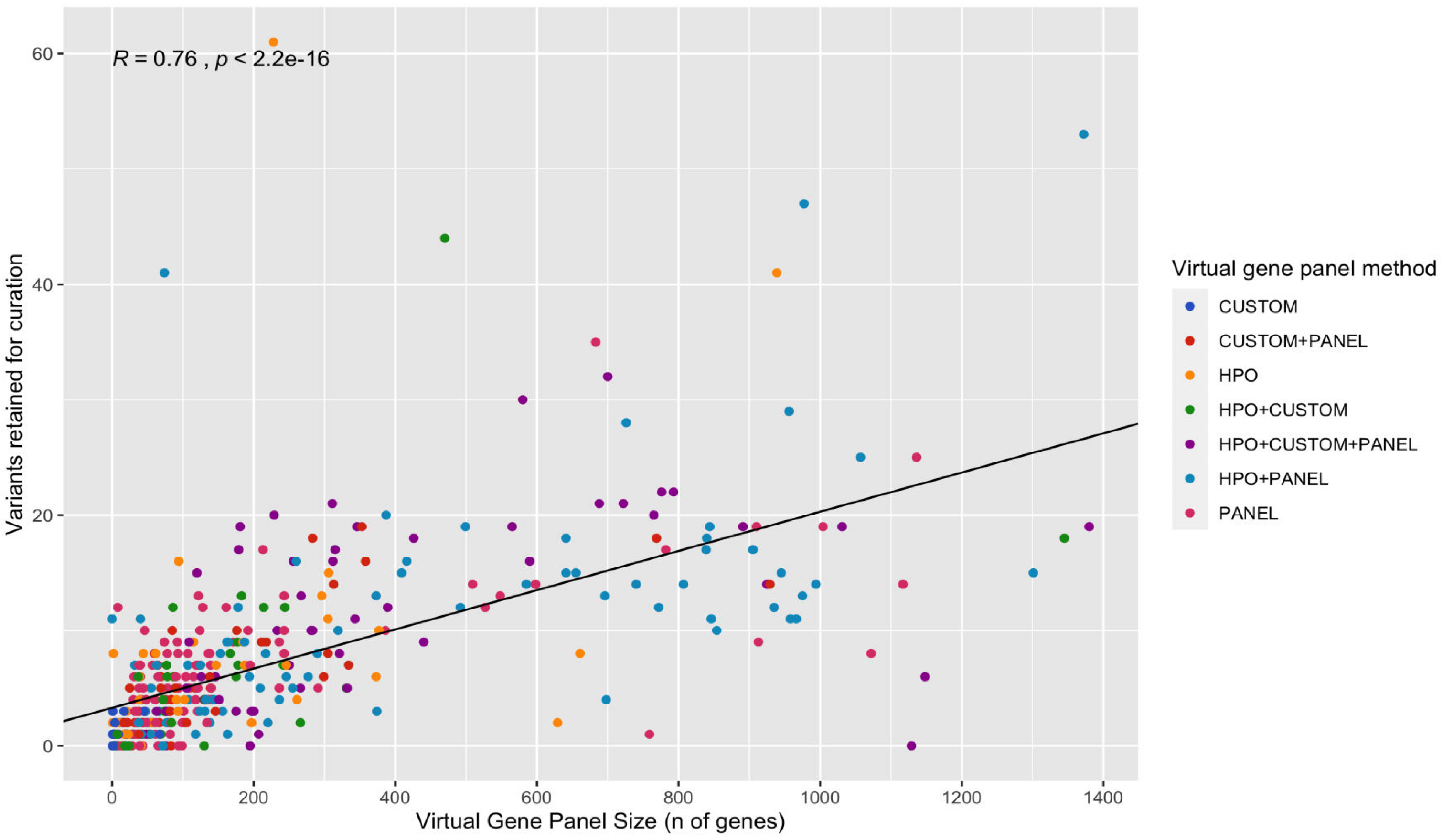

Supplementary Figure 4. Correlation between panel size and variants retained for clinical interpretation.

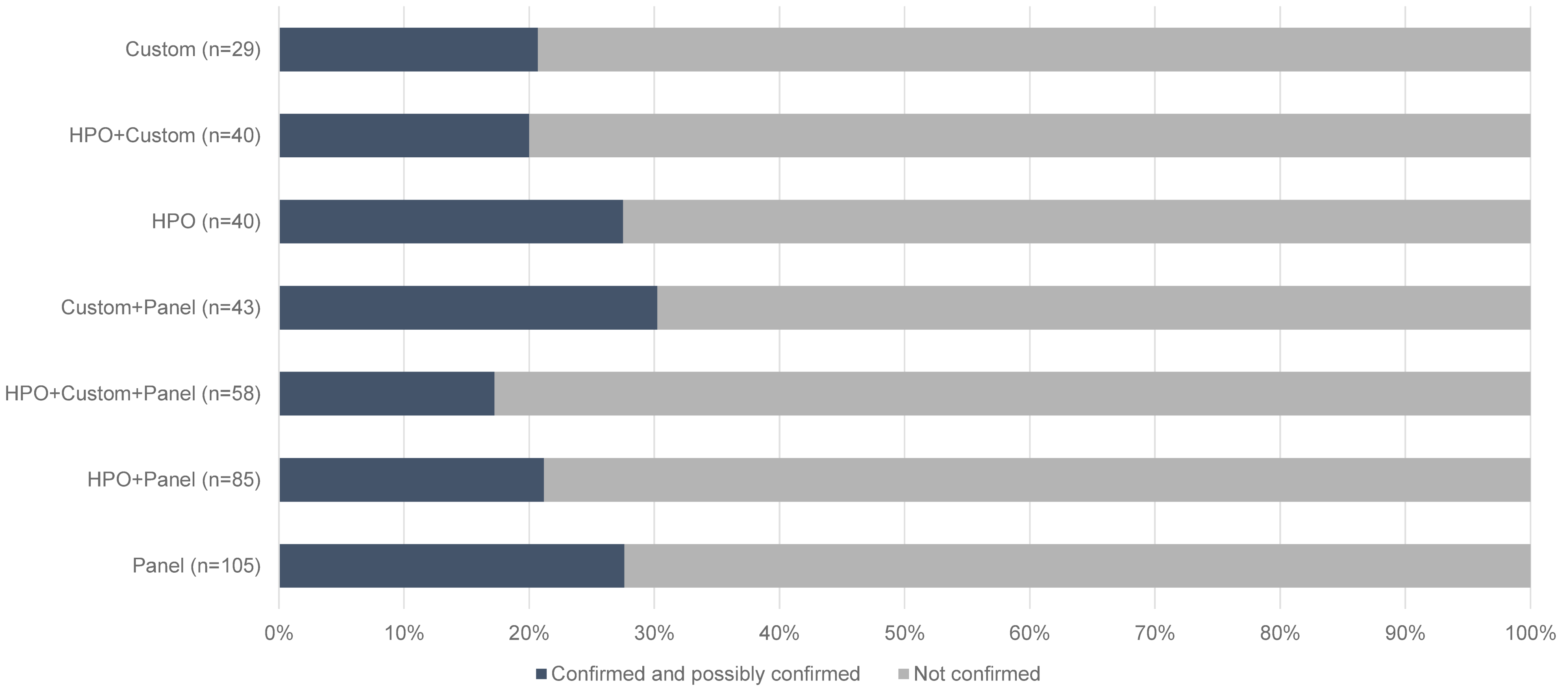

Supplementary Figure 5. Comparison of diagnostic rate among the different methods used for virtual gene panel generation. HPO- Human Phenotype Ontology
