# Supplementary material for: Personalised virtual gene panels reduce interpretation workload and maintain diagnostic rates of proband-only clinical exome sequencing for rare disorders
Source: J Med Genet. 2021 Apr 20;59(4):393–8. doi: 10.1136/jmedgenet-2020-107303 (PMC8961756; doi:10.1136/jmedgenet-2020-107303)
Supplement: Supplementary data [file jmedgenet-2020-107303supp003.pdf]

Case ID	Phenotype*	Gene symbol	Transcript	Nucleotide change	Protein change	Variant consequence	Variant Zygosity	ACMG Classification(1)	Gene inheritance	Novel at time of analysis (2)	References(3)
FE12015479	HP:0000316 - HYPERTELORISM<br>HP:0001250 - SEIZURES<br>HP:0001344 - ABSENT SPEECH<br>HP:0010864 - INTELLECTUAL DISABILITY, SEVERE<br>HP:0011081 - INCISOR MACRODONTIA<br>HP:0012120 - METHYLMALONIC ACIDURIA	*ACSF3*	NM_174917.3	c.1075G>A<br>c.1672C>T	p.(Glu359Lys)<br>p.(Arg558Trp)	missense<br>missense	Heterozygous<br>Heterozygous	4<br>4	AR	NO<br>NO	PMID: 21785126<br>PMID: 21841779
FE17012319	Late onset axonal neuropathy.<br>Nerve conduction studies show axonal motor and sensory neuropathy.	*AMPD1*	NM_000036.2	c.133C>T	p.(Gln45Ter)	nonsense	Homozygous	3	AR	NO	PMID: 1631143<br>PMID: 14499869<br>PMID: 16021918<br>PMID: 21686757<br>PMID: 29095874<br>PMID: 10918252<br>PMID: 18855224<br>PMID: 23300193<br>PMID: 25525159
FE15007224	HP:0000252 - MICROCEPHALY<br>HP:0000646 - AMBLYOPIA<br>HP:0001263 - GLOBAL DEVELOPMENTAL DELAY	*ASPM*	NM 018136.4	c.7782_7783delGA	p.(Lys2595SerfsTer6)	frameshift	Homozygous	5	AR	NO	PMID: 19028728
FE17012524	HP:0002076 - MIGRAINE;<br>HP:0040293 - RIGHT HEMIPLEGIA	*ATP1A2*	NM_000702.3	c.314G>A	p.(Gly105Glu)	missense	Heterozygous	3	AD	YES	_________
FE17012482	Changes suggestive of a macular dystrophy, central macular scars.<br>Electroretinogram with no evidence of widespread retinopathy.	*BEST1*	NM_004183.3	c.97T>C<br>c.152G>A	p.(Tyr33His)<br>p.(Arg51Lys)	missense<br>missense	Compound heterozygous<br>Compound heterozygous	4<br>4	AR	NO<br>YES	PMID: 25489231<br>_________
FE14001966	HP:0002721 - Oral ulcer<br>HP:0004315 - IGG DEFICIENCY<br>HP:0100633 - ESOPHAGITIS	*CARD11*	NM_032415.4	c.88C>G	p.(Arg30Gly)	missense	Heterozygous	4	AD,AR	YES	_________
FE11012584	Meckel-Gruber syndrome	*CEP290*	NM_025114.3	c.4825C>T	p.Gln1609Ter	nonsense	Homozygous	4	AR	YES	_________

Case ID	Phenotype*	Gene symbol	Transcript	Nucleotide change	Protein change	Variant consequence	Variant Zygosity	ACMG Classification(1)	Gene inheritance	Novel at time of analysis (2)	References(3)
FE16027107	HP:0000121 - NEPHROCALCINOSIS<br>HP:0002150 - HYPERCALCIURIA<br>HP:0002917 - HYPOMAGNESEMIA	*CLDN16*	NM_006580.3	c.445C>T	p.(Arg149Ter)	nonsense	Homozygous	5	AR	NO	PMID: 10390358<br>PMID: 25525159
FE16024957	Osteogenesis imperfecta	*COL1A2*	NM_000089.3	c.1378G>A	p.(Gly460Ser)	missense	Heterozygous	4	AD	NO	PMID: 8829649
FE16025631	Lethal skeletal dysplasia<br>suggestive of osteogenesis imperfecta.	*COL1A2*	NM_000089.3	c.2845G>A	p.(Gly949Ser)	missense	Heterozygous	4	AD	NO	PMID: 8081394
FE16019022	HP:0000252 - MICROCEPHALY<br>HP:0000518 - CATARACT<br>HP:0000545 - MYOPIA<br>HP:0002342 - INTELLECTUAL DISABILITY, MODERATE;<br>HP:0003756 - SKELETAL MYOPATHY	*COL6A2*	NM_058174.2	c.2548_2549delCT	p.(Leu850AspfsTer48)	frameshift	Heterozygous	3	AR, AD	YES	________
FE16017081	HP:0000006 - AUTOSOMAL DOMINANT<br>HP:0001417 - X-LINKED<br>HP:0003198 - MYOPATHY	*COL6A3*	NM_004369.3	c.3331G>A	p.(Ala1111Thr)	missense	Compound heterozygous	3	AR	NO	rs151021451
				c.1688A>G	p.(Asp563Gly)	missense	Compound heterozygous	3		YES	________
FE15009211	HP:0000384 - PREAURICULAR SKIN TAG<br>HP:0000639 - NYSTAGMUS<br>HP:0000703 - DENTINOGENESIS IMPERFECTA<br>HP:0000767 - PECTUS EXCAVATUM<br>HP:0000846 - ADRENAL INSUFFICIENCY<br>HP:0000953 - HYPERPIGMENTATION OF THE SKIN<br>HP:0001010 - HYPOPIGMENTATION OF THE SKIN<br>HP:0001510 - GROWTH DELAY<br>HP:0001903 - ANEMIA<br>HP:0007509 - PATCHY HYPO- AND HYPERPIGMENTATION<br>HP:0012368 - FLAT FACE<br>HP:0030021 - AURICULAR TAG	*COL7A1*	NM_000094.3	c.4867C>A	p.(Pro1623Thr)	missense	Compound heterozygous	4	AR	YES	________
				c.4172dupC	p.(Gly1392ArgfsTer10)	frameshift	Compound heterozygous	3		NO	PMID: 10504458
FE17000123	HP:0000426 - PROMINENT NASAL BRIDGE<br>HP:0001166 - ARACHNODACTYLY<br>HP:0001762 - TALIPES EQUINOVARUS<br>HP:0005684 - DISTAL ARTHROGRYPOSIS<br>HP:0012745 - SHORT PALPEBRAL FISSURE	*DSE*	NM_013352.2	c.1763A>G	p.(His588Arg)	missense	Homozygous	3	AR	YES	________

Case ID	Phenotype*	Gene symbol	Transcript	Nucleotide change	Protein change	Variant consequence	Variant Zygosity	ACMG Classification(1)	Gene inheritance	Novel at time of analysis (2)	References(3)
FE15019972	HP:0000175 - CLEFT PALATE<br>HP:0000347 - MICROGNATHIA<br>HP:0000973 - CUTIS LAXA<br>HP:0001382 - JOINT HYPERMOBILITY<br>HP:0002827 - HIP DISLOCATION<br>HP:0007522 - INCREASED NUMBER OF SKIN FOLDS	*FKBP14*	NM_017946.3.	c.362dupC	p.(p.Glu122ArgfsTer7)	frameshift	Homozygous	5	AR	NO	PMID: 22265013<br>PMID: 27905128<br>PMID: 28617417<br>PMID: 24677762
FE15004412	Imperforate anus.<br>Nerve conduction studies shows axonal motor and sensoty neuropathy	*GDAP1*	NM_018972.2	c.146T>C	p.(Leu49Ser)	missense	Homozygous	3	AR,AD	YES	_________
FE17007836	Mixed pattern of axonal degeneration<br>and demyelination.	*GJB1*	NM_001097642.2	c.145T>C	p.(Ser49Pro)	missense	Hemizygous	4	XLD	NO	PMID: 12207932
FE16002937	HP:0000054 - MICROPENIS<br>HP:0000175 - CLEFT PALATE<br>HP:0000204 - CLEFT UPPER LIP<br>HP:0000601 - HYPOTELORISM<br>HP:0000871 - PANHYPOPITUITARISM	*GLI2*	NM_005270.4	c.922delT	p.(Ser308Glnfs)	frameshift	Heterozygous	4	AD	YES	_________
FE17003778	HP:0002342 - INTELLECTUAL DISABILITY, MODERATE<br>HP:0007108 - DEMYELINATING PERIPHERAL NEUROPATHY	*HARS*	NM_002109.4	c.1190T>C	p.(Leu397Pro)	missense	Compound heterozygous	3	AR	YES	_________
				c.641G>A	p.(Arg214Gln)	missense	Compound heterozygous	3		YES	_________
FE16005088	HP:0000365 - HEARING IMPAIRMENT<br>HP:0000635 - BLUE IRIDES<br>HP:0001010 - HYPOPIGMENTATION OF THE SKIN<br>HP:0001270 - MOTOR DELAY<br>HP:0002211 - WHITE FORELOCK	*KITLG*	NM_000899.4	c.443T>C	p.(Ile148Thr)	missense	Homozygous	3	AR	NO	rs751013211
FE16010343	HP:0002415 - LEUKODYSTROPHY	*LAMA2*	NM_000426.3	c.1893_1897delCTTGA	p.(Asp631Glufs)	frameshift	Compound heterozygous	5	AR	NO	PMID: 11938437
				c.4307G>A	p.(Cys1436Tyr)	missense	Compound heterozygous	3		YES	_________
FE14009987	HP:0000609 - OPTIC NERVE HYPOPLASIA<br>HP:0001332 - DYSTONIA<br>HP:0006978 - DYSMYELINATING LEUKODYSTROPHY<br>Profound bilateral SNHL	*LHFPL5*	NM 182548.3	c.395G>A	p.(Trp132Ter)	missense	Homozygous	4	AR	YES	_________

Case ID	Phenotype*	Gene symbol	Transcript	Nucleotide change	Protein change	Variant consequence	Variant Zygosity	ACMG Classification(1)	Gene inheritance	Novel at time of analysis (2)	References(3)
FE17015560	Hypertelorism, sclerocornea, sacral dimple with a small skin appendage. 3MC syndrome	*MASP1*	NM_139125.3	c.542G>A	p.(Cys181Tyr)	missense	Homozygous	4	AR	YES	_________
FE17012304	HP:0001629 - VENTRICULAR SEPTAL DEFECT<br>HP:0001631 - ATRIAL SEPTAL DEFECT<br>HP:0001719 - DOUBLE OUTLET RIGHT VENTRICLE<br>HP:0006970 - PERIVENTRICULAR LEUKOMALACIA<br>HP:0007229 - INTRACEREBRAL PERIVENTRICULAR CALCIFICATIONS	*MED12*	NM_005120.2	c.5941A>G	p.(Asn1981Asp)	missense	Hemizygous	3	XLR	YES	_________
FE17022800	Significant hypotonia, no spontaneous respiratory drive, paucity of movements, pleural effusions.	*MTM1*	NM_000252.2	c.1189dupT	p.(Tyr397LeufsTer2)	frameshift	Hemizygous	4	XLR	YES	_________
FE16024727	Congenital Microvillus Atrophy	*MYO5B*	NM_001080467.2	c.4739_4740delAC	p.(His1580LeufsTer3)	frameshift	Homozygous	4	AR	YES	_________
FE16008058	HP:0002652 - SKELETAL DYSPLASIA;<br>HP:0004322 - SHORT STATURE	*MYO5B*	NM_001080467.2	c.673C>T	p.(Gln225Ter)	nonsense	Compound heterozygous	4	AR	YES	_________
				c.1355A>G	p.(Gln452Arg)	missense	Compound heterozygous	3		YES	_________
FE10011560	Polydactyly, short limbs, cleft lip, micrognathia, narrow thorax.	*NEK1*	NM_001199397.1	c.1014_1015delTA	p.(His338GlnfsTer22)	frameshift	Homozygous	4	AR	YES	_________
FE16022090	Multiple syndromes,<br>nonrelated symphalangism spectrum disorder	*NOG*	NM_005450	c.124C>A	p.(Pro42Thr)	missense	Heterozygous	4	AD	NO	PMID: 23732071
FE15011757	HP:0004322 - SHORT STATURE	*OBSL1*	NM_015311.2	c.2100_2122del	p.(Gly701HisfsTer31)	frameshift	Homozygous	4	AR	YES	_________

Case ID	Phenotype*	Gene symbol	Transcript	Nucleotide change	Protein change	Variant consequence	Variant Zygosity	ACMG Classification(1)	Gene inheritance	Novel at time of analysis (2)	References(3)
FE16007307	HP:0000726 - DEMENTIA<br>HP:0000727 - FRONTAL LOBE DEMENTIA<br>HP:0001251 - ATAXIA<br>HP:0002415 - LEUKODYSTROPHY	*PDGFB*	NM_002608.2	c.295A>G	p.(Thr99Ala)	missense	Heterozygous	3	AD	YES	_________
FE17012696	Large ventricles with cystic changes bilaterally, low signal thalami and basal ganglia, white matter abnormalities and small cerebellum.	*PDHA1*	NM_000284.3	c.999_1008+10del	p.(Glu334LysfsTer6)	frameshift	Heterozygous	4	XLD	YES	_________
FE17005848	HP:0000110 - RENAL DYSPLASIA<br>HP:0000803 - RENAL CORTICAL CYSTS<br>HP:0001562 - OLIGOHYDRAMNIOS	*PKHD1*	NM_138694.3	c.10315delG	p.(Asp3439MetfsTer6)	frameshift	Homozygous	5	AR	YES	_________
FE14022150	HP:0000256 - MACROCEPHALY<br>HP:0001548 - OVERGROWTH	*PTCH1*	NM_000264.3	c.1067+1G>T	p.?	splicing	Heterozygous	5	AD	YES	_________
FE15024053	HP:0001251 - ATAXIA	*SACS*	NM_014363.5	c.10906C>T	p.(Arg3636Ter)	nonsense	Homozygous	4	AR	NO	PMID: 18465152
FE16021083	HP:0001263 - GLOBAL DEVELOPMENTAL DELAY<br>HP:0001290 - GENERALIZED HYPOTONIA<br>HP:0002789 - TACHYPNEA<br>HP:0007308 - EXTRAPYRAMIDAL DYSKINESIA	*SLC16A2*	NM_006517.4	c.644C>T	p.(Pro215Leu)	missense	Hemizygous	4	XLD	NO	PMID: 27620904
FE16010798	Segmental, tremulous dystonia	*SLC20A2*	NM_006749.4	c.211C>T	p.(Arg71Cys)	missense	Heterozygous	3	AD	YES	_________
FE16019783	HP:0000776 - CONGENITAL DIAPHRAGMATIC HERNIA<br>HP:0001338 - PARTIAL AGENESIS OF THE CORPUS CALLOSUM<br>HP:0001511 - INTRAUTERINE GROWTH RETARDATION<br>HP:0001643 - PATENT DUCTUS ARTERIOSUS<br>HP:0002650 - SCOLIOSIS<br>HP:0002937 - HEMIVERTEBRAE<br>HP:0003808 - ABNORMAL MUSCLE TONE	*SMARCB1*	NM_003073.3	c.1129C>T	p.(Arg377Cys)	missense	Heterozygous	3	AD	YES	_________

Case ID	Phenotype*	Gene symbol	Transcript	Nucleotide change	Protein change	Variant consequence	Variant Zygosity	ACMG Classification(1)	Gene inheritance	Novel at time of analysis (2)	References(3)
FE10011847	HP:0001256 - INTELLECTUAL DISABILITY, MILD<br>HP:0001332 - DYSTONIA	*SPG11*	NM 025137.3	c.3319_3322delTGTT	p.(Cys1107Ter)	nonsense	Homozygous	4	AR	YES	_________
FE16026183	HP:0001348 - BRISK REFLEXES<br>HP:0002313 - SPASTIC PARAPARESIS	*SPTAN1*	NM_001130438.2	c.4737C>G	p.(Tyr1579Ter)	nonsense	Heterozygous	3	AD	YES	_________
FE16021580	Axonal neuropathy	*SPTLC1*	NM 001281303.1	c.859C>T	p.(Arg287Ter)	nonsense	Heterozygous	4	AD	YES	_________
FE16026525	HP:0008504 - MODERATE SENSORINEURAL HEARING IMPAIRMENT	*STRC*	NM_153700.2	c.4402C>T	p.(Arg1468Ter)	nonsense	Compound heterozygous	5	AR	NO	PMID: 26011646
				c.4517T>C	p.(Leu1506Pro)	missense	Compound heterozygous	3		YES	_________
FE16024864	HP:0000243 - TRIGONOCEPHALY<br>HP:0004467 - PREAURICULAR PIT<br>HP:0011218 - ABNORMAL SHAPE OF THE FRONTAL REGION	*TFAP2A*	NM_003220.2	c.746T>A	p.(Leu249Gln)	missense	Heterozygous	4	AD	YES	_________
FE16024987	HP:0001337 - AKINETIC RIGID<br>HP:0003674 - ONSET	*TH*	NM_199292.2	c.440G>A	p.(Arg147Gln)	missense	Homozygous	3	AR	YES	_________
FE16010668	Meckel Gruber syndrome, multicystis dysplastic kidneys and posterior fossa abnormalities.<br>Postmortem findings of meningocele, mild ventriculomegaly and some liver changes.	*TMEM67*	NM_153704.5	c.2522A>C	p.(Gln841Pro)	missense	Compound heterozygous	4	AR	NO	PMID: 19574260<br>PMID: 26092869<br>PMID: 28508964<br>PMID: 28497568<br>PMID: 27434533
				c.548C>A	p.(Ser183Tyr)	missense	Compound heterozygous	3		YES	_________
FE15010400	HP:0001251 - ATAXIA<br>HP:0001270 - MOTOR DELAY<br>HP:0002058 - MYOPATHIC FACIES<br>[illegible]	*TMEM67*	NM_153704.5	c.869+1G>C	p.?	splicing	Compound heterozygous	4	AR	YES	_________

Case ID	Phenotype*	Gene symbol	Transcript	Nucleotide change	Protein change	Variant consequence	Variant Zygosity	ACMG Classification(1)	Gene inheritance	Novel at time of analysis (2)	References(3)
	HP:0002419 - MOLAR TOOTH SIGN ON MRI<br>HP:0011398 - CENTRAL HYPOTONIA			c.894T>G	p.(Phe298Leu)	missense	Compound heterozygous	3		YES	_________
FE16001356	HP:0000510 - ROD-CONE DYSTROPHY<br>HP:0001513 - OBESITY<br>HP:0001644-DILATED CARDIOMYOPATHY.	*ALMS1*	NM_015120.4	c.10769delC	p.(Thr3590Lysfs)	frameshift	Homozygous	5	AR	NO	PMID: 31810438
FE15001890	Dysplastic left cerebral hemisphere. Malformation of cortex and white matter. Absent corpus callosum and small cerebellum, interhemispheric cyst. Syndactyly left hand and toes. Cysts on tongue. Mild developmental delay.	*OFD1*	NM_003611.2	c.1411+1G>A het	p.?	splicing	Heterozygous	5	XLD	YES	_________
FE17027804	Ventriculomegaly, hypotonia, pachygyria and abnormal posterior fossa structures, small cerebellum and pons. Suspected dystroglycanopathy.	*POMT1*	NM_001080522.2	c.704G>T	p.(Gly235Val)	missense	Homozygous	3	AR	YES	_________
FE15011079	HP:0000175-CLEFT PALATE<br>HP:0000280-COARSE FACIAL FEATURES	*CDKN1C*	NM_001362474	c.721C>T	p.(Gln241Ter)	nonsense	Heterozygous	4	AD	NO	PMID: 11414765
FE17012633	HP:0002804-ARTHROGRYPOSIS MULTIPLEX CONGENITA	*ECEL1*	NM_004826.2	c.1184+2T>A	p.?	splicing	Heterozygous	5	AR	YES	_________
				c.491T>C	p.(Leu164Pro)	missense	Heterozygous	3		YES	_________
FE17023866	Likely clinical diagnosis of Larsen syndrome.	*FLNB*	NM_001164317.1	c.535G>A	p.(Ala179Thr)	missense	Heterozygous	3	AD	YES	_________
FE17012318	Congenital myopathy and scoliosis	*SCN4A*	NM_000334.4	c.1342A>G	p.(Met448Val)	missense	Heterozygous	3	AD	YES	_________

Case ID	Phenotype*	Gene symbol	Transcript	Nucleotide change	Protein change	Variant consequence	Variant Zygosity	ACMG Classification(1)	Gene inheritance	Novel at time of analysis (2)	References(3)
FE17029030	Encephalopathic event with bilateral symmetrical basal ganglia signal change and brainstem haemorrhage/necrosis.	*RANBP2*	NM_006267.4.	c.1754C>T	p.(Thr585Met)	missense	Heterozygous	5	AD	NO	PMID: 19118815<br>PMID: 25522933
FE17013473	Nasal alae erythema, malar rash, laryngeal and supraglottic ulceration, two elevated interferon signatures. Normal development.	*PSTPIP1*	NM_003978.3	c.247C>T	p.(Gln83Ter)	nonsense	Heterozygous	3	AD	YES	_________
FE17030781	Hereditary Spastic Paraplegia	*SPAST*	NM_014946.3	c.1115G>C	p.(Arg372Thr)	missense	Heterozygous	5	AD	NO	_________
FE16000345	Early onset dementia, likely autosomal dominant.	*PSEN1*	NM_000021.3	c.737C>A	p.(Ala246Glu)	missense	Heterozygous	5	AD	NO	PMID: 7596406
FE17022955	Early onset epileptic encephalopathy, facial dysmorphisms, hypotonia and vacuolating leucodystrophy.	*ADSL*	NM_000026.2	c.1277G>A	p.(Arg426His)	missense	Heterozygous	5	AR	NO	PMID 10090474
				c.632T>A	p.(Leu211His)	missense	Heterozygous	3		YES	_________
FE18001063	Episodic ataxia. Probably type 2 with some chronic ataxia developing in later life. 3 affected individuals alive.	*CACNA1A*	NM_001127221.1	c.6047G>A	p.(Gly2016Glu)	missense	Heterozygous	3	AD	NO	rs16024
FE16026207	HP:0001251-CEREBELLAR ATAXIA	*AFG3L2*	NM_006796.2	c.2069G>A	p.(Ser690Asn)	missense	Heterozygous	3	AD	YES	_________
FE18002417	Clinical diagnosis of familial hemiplegic migraine	*ATP1A2*	NM_000702.3	c.2563G>A	p.(Gly855Arg)	missense	Heterozygous	4	AD	NO	PMID: 24921013

Case ID	Phenotype*	Gene symbol	Transcript	Nucleotide change	Protein change	Variant consequence	Variant Zygosity	ACMG Classification[1]	Gene inheritance	Novel at time of analysis [2]	References[3]
FE17027226	Severe demyelinating neuropathy. Presumed X linked CMT	*GJB1*	NM_000166.6	c.392T>C	p.(Leu131Pro)	missense	Hemizygous	4	XLD	NO	rs1555937166<br>PMID: 9818870<br>PMID: 27804109
FE17026201	Clinical diagnosis of primary ciliary dyskinesia.	*HYDIN*	NM_001270974.1.	c.10441C>T	p.(Arg3481Trp)	missense	Heterozygous	3	AR	NO	rs76078590
				c.9347C>G	p.(Thr3116Arg)	missense	Heterozygous	3		YES	_________
				c.6827A>G	p.(Gln2276Arg)	missense	Heterozygous	3		YES	_________
FE17017837	HP:0000252-MICROCEPHALY<br>HP:0000518-CATARACT<br>HP:0002197-GENERALIZED SEIZURES<br>HP:0002342-INTELLECTUAL DISABILITY, MODERATE<br>HP:0003508-PROPORTIONATE SHORT STATURE	*DYNC1H1*	NM 014946.3	c.7874C>G	p.(Ala2625Gly)	missense	Heterozygous	3	AD	YES	_________
FE17005729	Proximal muscle weakness. Query Limb Girdle muscular dystrophy	*ANO5*	NM_213599.2	c.692G>T	p.(Gly231Val)	missense	Compound heterozygous	4	AR	NO	PMID: 20096397<br>PMID: 23041008
				c.191dupA	p.(Asn64LysfsTer15)	frameshift	Compound heterozygous	5		NO	PMID: 23606453<br>PMID: 27708273
FE17014128	Joubert syndrome. Muscular hypotonia, Motor delay, Oculomotor apraxia. Metabolic screening revealed isolated Orotic aciduria, suggesting UMPS deficiency.	*UMPS*	NM_000373.3	c.928T>G	p.Phe310Val Hom	missense	Homozygous	3	AR	YES	_________
FE10005509	Profound generalised muscle weakness, severe cognitive impairment, inflammatory bowel disease, recurrent renal calculi.	*SLC5A7*	NM_021815.2	c.1240T>A	p.(Tyr4l4Asn)	missense	Homozygous	3	AR	YES	_________
FE17025791	End stage renal failure at about 6 months old. Appearance of small bright kidneys bilaterally (hypodysplasia). Previous likely unilateral hydronephrosis. Appearance of rhizomelia and brachydactyly. Cone shaped epiphysis. .Query Skeletal Ciliopathy	*WDR35*	NM_001006657.1	c.392G>T	p.(Cys131Phe)	missense	Homozygous	3	AR	YES	_________
FE17022845AV	Respiratory distress, hypoxia, required extensive resuscitation, multiple thromboses, acute kidney injury.	*WT1*	NM_024426.4	c.1096C>T	p.(Arg366Cys)	missense	Heterozygous	4	AD	NO	PMID: 9529364<br>doi.org/10.1002/ajmg.a.31924

Case ID	Phenotype*	Gene symbol	Transcript	Nucleotide change	Protein change	Variant consequence	Variant Zygosity	ACMG Classification(1)	Gene inheritance	Novel at time of analysis (2)	References(3)
FE18004080	Born with distal arthrogryposis. Normal IQ.	*PIEZO2*	NM 022068.2.	c.5951T>C	p.(Phe198Ser)	missense	Heterozygous	3	AD	NO	rs1408124992
FE17031083	Failure to thrive, hypotonia, global delay, microcephaly, telecanthus/epicanthic folds, long philtrum tented upper lip. High or undescended testes small genitalia, deep foot creases. Dystonia.	*GBE1*	NM_000158.4	c.721A>G	p.(Met241Val)	missense	Heterozygous	3	AR	NO	rs747155575
		GBE1	NM_000158.4	c.691+2T>C	p.?	splicing	Heterozygous	4		NO	rs192044702
FE15018880	HP:0000047-HYPOSPADIAS<br>HP:0000316-HYPERTELORISM<br>HP:0000358-POSTERIORLY ROTATED EARS<br>HP:0002564-CONGENITAL HEART DISEASE<br>HP:0008751-LARYNGEAL CLEFT	*MID1*	NM_000381.3	c.1286G>T	p.Ser429Ile	missense	Heterozygous	3	AD	YES	__________
FE18003789	Possible diagnosis of hereditary. Motor and sensory neuropathy. Pes cavu, nerve conduction study showed mixed motor and sensory axonal type polyneuropathy.	*MFN2*	NM_014874.3	c.838C>T	p.(Arg280Cys)	missense	Heterozygous	4	AD	YES	__________
FE18010010	HP:0002633-VASCULITIS<br>HP:0002841-RECURRENT FUNGAL INFECTIONS<br>HP:0010885-AVASCULAR NECROSIS	*TREX1*	NM_033629.4.	c.814delG	p.(Asp272IlefsTer5)	frameshift	Heterozygous	4	AD	YES	__________
FE14012423	HP:0000028-CRYPTORCHIDISM<br>HP:0000187-BROAD ALVEOLAR RIDGES<br>HP:0000252-MICROCEPHALY<br>HP:0000586-SHALLOW ORBITS<br>HP:0001272-CEREBELLAR ATROPHY<br>HP:0002342-INTELLECTUAL DISABILITY, MODERATE<br>HP:0003508-PROPORTIONATE SHORT STATURE<br>HP:0008551-MICROTIA	*ORC6*	NM_014321.3	c.71C>T	p.Ala24Val	missense	Homozygous	3	AR	YES	__________
FE11700617	HP:0000347-MICROGNATHIA<br>HP:0000494-DOWNSLANTED PALPEBRAL FISSURES<br>HP:0000625-EYELID COLOBOMA<br>HP:0000676-ABNORMALITY OF THE INCISOR<br>HP:0011330-METOPIC SYNOSTOSIS<br>HP:0040080-ANTEVERTED EARS<br>HP:0200102-SPARSE OR ABSENT EYELASHES	*TCOF1*	NM_001135243.1	c.1444_1459dup	p.(Glu487ValfsTer4)	frameshift	Heterozygous	4	AD	YES	__________
FE16002792	Childhood onset, progressive sensorineural hearing loss, cochlear implant user. Family history of SNHL.	*TMPSSR3*	NM_024022.2	c.1276G>A	p.(Ala426Thr)	missense	Heterozygous	3	AR	NO	rs56264519<br>PMID: 21786053<br>PMID: 28566687
		TMPSSR3	NM_024022.2	c.208delC	p.(His70ThrfsTer19)	frameshift	Heterozygous	5	AR	NO	PMID 28566687<br>PMID 11907649<br>PMID 29293505

Case ID	Phenotype*	Gene symbol	Transcript	Nucleotide change	Protein change	Variant consequence	Variant Zygosity	ACMG Classification(1)	Gene inheritance	Novel at time of analysis (2)	References(3)
FE18013538	Possible Joubert syndrome. Hypotonia, disordered breathing, developmental delay, nystagmus and the appearance of a molar tooth sign on MRI scan.	*AHI1*	NM_017651.4	c.2296G>A	p.(Gly766Arg)	missense	Homozygous	4	AR	YES	__________
FE17024387	HP:0001188-HAND CLENCHING<br>HP:0005484-POSTNATAL MICROCEPHALY<br>HP:0007340-LOWER LIMB MUSCLE WEAKNESS<br>HP:0008780-CONGENITAL BILATERAL HIP DISLOCATION<br>HP:0010864-INTELLECTUAL DISABILITY, SEVERE	*CHD8*	NM_001170629.1	c.2493C>G	p.(Asn831Lys)	missense	Heterozygous	3		YES	__________
FE17020997	HP:0001093-OPTIC NERVE DYSPLASIA<br>HP:0008619-BILATERAL SENSORINEURAL HEARING IMPAIRMENT	*CHD7*	NM_017780.3	c.3089A>G	p.(Asn1030Ser)	missense	Heterozygous	4	AD	NO	PMID 21041284<br>PMID 25077900
FE15011417	Multiple cavernous malformations, learning difficulties, previous absences, multiple naevi, multicystic dysplastic kidneys. Parental LD.	*PDCD10*	NM_145860.1.	c.496G>T	p.(Glu166Ter)	nonsense	Heterozygous	5	AD	NO	rs1559944602
FE16001689	Moderate sensorineural hearing loss.	*PDZD7*	NM_001195263.1.	c.1648C>T	p.(Gln550Ter)	nonsense	Homozygous	5	AR	NO	rs1554834161<br>PMID 26849169<br>PMID 20440071
FE9010089	HP:0005360-SUSCEPTIBILITY TO CHICKENPOX	*CTPS1*	NM_001905.3	c.1714_1724del	p.(Gly572ProfsTer2)	frameshift	Homozygous	3	AR	YES	__________
FE18010049	HP:0001040-MULTIPLE PTERYGIA	*CHRNA1*	NM_000079.3.	c.685C>T	p.(Arg229Cys)	missense	Homozygous	3	AR	NO	PMID 23037934
FE18001423	Global developmental delay<br>Facial dysmorphic features<br>?Sotos syndrome	*C5orf42*	NM_023073.3.	c.98T>C<br>c.3341T>A	p.(Phe33Ser)<br>p.(Val1114Glu)	missense<br>missense	Heterozygous<br>Heterozygous	3<br>3	AR	YES<br>YES	__________<br>__________

Case ID	Phenotype*	Gene symbol	Transcript	Nucleotide change	Protein change	Variant consequence	Variant Zygosity	ACMG Classification(1)	Gene inheritance	Novel at time of analysis (2)	References(3)
FE17013163	HP:0000752-HYPERACTIVITY<br>HP:0001007-HIRSUTISM<br>HP:0010864-INTELLECTUAL DISABILITY, SEVERE<br>San filippo syndrome	*HGSNAT*	NM_152419.2	c.744-2A>G	p.?	splicing	homozygous	4	AR	NO	PMID 19479962
FE18023423	Renal tubular acidosis, keratopathy	*SLC4A4*	NM_003759.3.	c.691_692delinsTA	p.(Met231Ter)	nonsense	Heterozygous	4	AR	YES	__________
FE18023071	Onset in teens of aching muscles after minor exercise. EMG reported borderline myopathic changes. Mild generalised weakness in all 4 limbs, creatine kinase levels normal.	*ANO5*	NM_213599.2	c.692G>T	p.(Gly231Val)	missense	Heterozygous	4	AR	NO	PMID 20096397
FE18032492	HP:0004345-GANGLIOSIDE ACCUMULATION	*PMM2*	NM_000303.2.	c.367C>T	p.(Arg123Ter)	nonsense	Heterozygous	5	AR	NO	rs191295403<br>PMID 11058895<br>PMID 12705494<br>PMID 17166182
				c.722G>C	p.(Cys241Ser)	missense	Heterozygous	5		NO	PMID 11715002<br>PMID 22012410<br>PMID 28566178<br>PMID 28425223<br>PMID 29482223
FE18021754	Severe global developmental delay, facial dysmorphism, congenital cataracts, epilepsy.	*MED25*	NM_030973.3.	c.518T>C	p.(Ile173Thr)	missense	Homozygous	4	AR	NO	PMID 30800049
FE18021035	Hereditary spastic paraparesis, positive family history.	*SPAST*	NM_014946.3	c.286delG	p.(Ala96ArgfsTer65)	frameshift	Heterozygous	5	AD	NO	PMID 11015453
FE11012997	Progressive relatively severe demyelinating CMT.	*MPV17*	NM_002437.5	c.122G>A	p.(Arg4lGln)	missense	Homozygous	5	AR	NO	PMID 26437932

Case ID	Phenotype*	Gene symbol	Transcript	Nucleotide change	Protein change	Variant consequence	Variant Zygosity	ACMG Classification(1)	Gene inheritance	Novel at time of analysis (2)	References(3)
FE18011009	HP:0000196-LOWER LIP PIT<br>HP:0000492-ABNORMAL EYELID MORPHOLOGY<br>HP:0001849-FOOT OLIGODACTYLY<br>HP:0009099-MEDIAN CLEFT PALATE<br>HP:0009756-POPLITEAL PTERYGIUM	*IRF6*	NM_006147.4	c.250C>T	p.(Arg84Cys)	missense	Heterozygous	5	AD	NO	PMID 19036739
FE14014016	HP:0000717-AUTISM<br>HP:0000824-GROWTH HORMONE DEFICIENCY<br>HP:0002342-INTELLECTUAL DISABILITY, MODERATE<br>HP:0010627-ANTERIOR PITUITARY HYPOPLASIA	*ASH1L*	NM_018489.2.	c.6826C>T	p.(Arg2276Ter)	nonsense	Heterozygous	3	AD	YES	__________
FE18018059	HP:0001332-DYSTONIA<br>HP:0002180-NEURODEGENERATION<br>HP:0002454-EYE OF THE TIGER ANOMALY OF GLOBUS PALLIDUS<br>HP:0030890-HYPERINTENSITY OF CEREBRAL WHITE MATTER ON MRI	*PANK2*	NM_153638.3	c.629-2A>G	p.?	splicing	Homozygous	4	AR	NO	PMID 22930366
FE18024723	Prenatal pericardial effusion, fetal anaemia. respiratory distress at birth, seizures and abnormal EEG. MRI brain normal.	*RMND1*	NM_017909.3	c.87delG	p.(Met29IlefsTer2)	frameshift	Heterozygous	4	AR	YES	__________
FE18015666	HP:0001249-INTELLECTUAL DISABILITY<br>HP:0001387-JOINT STIFFNESS<br>HP:0012722-HEART BLOCK	*ANKRD11*	NM_001256182	c.7470+2T>A	p.?	splicing	Heterozygous	3	AD	YES	__________
FE18024073	HP:0000407-SENSORINEURAL DEAFNESS<br>HP:0000545-MYOPIA<br>HP:0003198-MYOPATHY	*CEP78*	NM_001098802.1	c.1450C>T	p.(Arg484Ter)	nonsense	Homozygous	4	AR	YES	__________
		ETFDH	NM_04453.3	c.1448C>T	p.(Pro483Leu)	missense	Homozygous	3	AR	NO	PMID 17584774<br>PMID 29530532
FE13004802	Severe bilateral sensorineural hearing loss.	*CABP2*	NM_001318496.1.	c.292C>T	p.(Arg98Ter)	nonsense	Homozygous	4	AR	NO	rs761766884
FE14007468	Moderate-severe learning disability, palmoplantar keratoderma, microcephaly	*PACS1*	NM_018026.3.	c.607C>T	p.(Arg203Trp)	missense	Heterozygous	5	AD	NO	PMID 23159249

Case ID	Phenotype*	Gene symbol	Transcript	Nucleotide change	Protein change	Variant consequence	Variant Zygosity	ACMG Classification(1)	Gene inheritance	Novel at time of analysis (2)	References(3)
FE18029719	Congenital unilateral cleft lip and palate along with lower lip pits, likely diagnosis of Van der Woude syndrome.	*IRF6*	NM_006147.4	c.1169T>C	p.(Ile390Thr)	missense	Heterozygous	3	AD	YES	__________
FE18030683	Non-syndromic severe bilateral congenital hearing loss.	*LOXHD1*	NM_144612.6	c.6306T>G	p.(Tyr2102Ter)	nonsense	Heterozygous	3	AR	YES	__________
				c.3727C>T	p.(Arg1243Trp)	missense	Heterozygous	3		NO	rs369695848
FE18030610	Learning difficulties, thin corpus callosum.	*SPG11*	NM_025137.3	c.5399_5402delins8	p.(Gln1800LeufsTer31)	frameshift	Homozygous	4	AR	NO	PMID 28237315
FE18029416	HP:0000308-MICRORETROGNATHIA<br>HP:0000581-BLEPHAROPHIMOSIS<br>HP:0000647-SCLEROCORNEA<br>HP:0001545-ANTERIORLY PLACED ANUS	*UBE3B*	NM_130466.3	c.469C>T	p.(Arg157Ter)	nonsense	Heterozygous	4	AR	YES	__________
				c.730C>T	p.(Gln244Ter)	nonsense	Heterozygous	4		YES	__________
FE18023357	Tylosis affecting his palmar and plantar surfaces, esophagitis.	*DSG1*	NM_001942.3	c.430A>T	p.(Arg144Ter)	nonsense	Heterozygous	5	AD	NO	PMID 19558595
FE18013680	Developmental delay , synophrys, patchy T2 white matter, hyperintensity in subcortical white matter.	*BCL11A*	NM_022893.3	c.192delC	p.(Ile64MetfsTer14)	frameshift	Heterozygous	5	AD	YES	__________
FE18031525	Low immunoglobulins, low vaccine response CVID, splenomegaly, coeliac, hyperthyroid, endometriosis and abdominal adhesions.	*STAT1*	NM 007315.3.	c.796G>A	p.(Val266Ile)	missense	Heterozygous	3	AD/AR	NO	PMID 26513235<br>PMID 23534974<br>PMID 26038974<br>PMID 29077208
FE18031312	HP:0001102-ANGIOID STREAKS OF THE FUNDUS	*ABCC6*	NM_001171.5	c.3490C>T	p.(Arg1164Ter)	nonsense	Heterozygous	5	AR	NO	PMID 10835642
				c.3421C>T	p.(Arg1141Ter)	nonsense	Heterozygous	5		NO	PMID 15086542<br>PMID 12714611<br>PMID 11179012<br>PMID 10954200

Case ID	Phenotype*	Gene symbol	Transcript	Nucleotide change	Protein change	Variant consequence	Variant Zygosity	ACMG Classification(1)	Gene inheritance	Novel at time of analysis (2)	References(3)
FE18000260	Macrophage activation syndrome, juvenile idiopathic arthritis.	*NLRP12*	NM_144687.3	c.2828_2829dupTC	p.(Arg944SerfsTer6)	frameshift	Heterozygous	3	AD	NO	rs533054990
FE14003054	Moderate sensorineural hearing loss.	*MARVELD2*	NM_001038603.2	c.1098G>A	p.(Trp366Ter)	nonsense	Homozygous	4	AR	NO	rs773552728
FE18030121	Multiple venous malformations	*GLMN*	NM_053274.2	c.157_161delAAGAA	p.(Lys53Ter)	nonsense	Heterozygous	5	AD	NO	rs762515373
FE13000175	HP:0001631-ATRIAL SEPTAL DEFECT<br>HP:0001642-PULMONARY STENOSIS<br>HP:0001647-BICUSPID AORTIC VALVE	*TAB2*	NM_015093.5.	c.1273C>T	p(Gln425Ter)	nonsense	Heterozygous	4	AD	YES	__________
FE18019948	Bilateral mild sensorineural hearing loss<br>Bilateral enlarged vestibular aqueducts	*SLC26A4*	NM_000441.1	c.1151A>G<br>c.1246A>C	p.(Glu384Gly)<br>p.(Thr416Pro)	missense<br>missense	Heterozygous<br>Heterozygous	5<br>5	AR	NO<br>NO	PMID 9618167<br>PMID 12788906<br>PMID 24224479<br>PMID 9618167<br>PMID 12788906<br>PMID 24224479
FE18026000	HP:0001903-ANAEMIA<br>HP:0001943-HYPOGLYCAEMIA<br>HP:0002242-ENTEROPATHY<br>HP:0003256-COAGULOPATHY	*ALG8*	NM_024079.4	c.1579T>A	p.(Ter527Argext*?)	LOF	Homozygous	5	AR	NO	rs1346281230
FE18031582	HP:0002652-SKELETAL DYSPLASIA	*SOX9*	NM_000346.3.	c.343T>A	p.(Trp115Arg)	missense	Heterozygous	3	AD	YES	__________
FE19002770	Child of consanguinous roma gypsy couple (? relationship)<br>Profound developmental delay, profound bilateral sensorineural hearing loss, progressive retinopathy based on worsening of ERGS and very limited vision, optic atrophy, seizures onset at 18 months, dystonic episodes, progressive scoliosis, marked hirsutism, hypoplastic basal ganglia, thin corpus callosum, decreased white matter.	*BSND*	NM_057176.2	c.23G>A	p.(Arg8Gln)	missense	Homozygous	3	AR	NO	PMID 29986705

Case ID	Phenotype*	Gene symbol	Transcript	Nucleotide change	Protein change	Variant consequence	Variant Zygosity	ACMG Classification(1)	Gene inheritance	Novel at time of analysis (2)	References(3)
FE18021366	Possible Walker-Warburg, severe ventriculomegaly, delayed sulcal and gyral formation , cerebellar vermis hypoplasia.	*TUBA1A*	NM_006009.3	c.652G>A	p.(Asp218Asn)	missense	Heterozygous	4	AD	NO	PMID 20466733
FE19003044	Congenital agammaglobulinaemia and absent B cells, macrocytosis, recurrent rhinitis, lichen, sclerosis.	*TCF3*	NM_001136139.2	c.1663G>A	p.(Glu555Lys)	missense	Heterozygous	5	AD	NO	PMID 24216514
FE19001679	Microcephaly, learning difficulties, possible hearing loss, flat retina, chiasmal hypoplasia.	*KIF11*	NM_004523.3	c.1129-1G>C	p.?	splicing	Heterozygous	5	AD	YES	__________
FE19003614	HP:0005208-SECRETORY DIARRHEA	*GUCY2C*	NM_ 004963.3.	c.2536G>A	p.(Val846Met)	missense	Heterozygous	3	AD	YES	__________
FE19000072	HP:0001181-ADDUCTED THUMBS<br>HP:0005684-DISTAL ARTHROGRYPOSIS<br>HP:0010557-OVERLAPPING FINGERS	*ECEL1*	NM_004826.3.	c.589G>A	p.(Gly197Ser)	missense	Homozygous	4	AR	NO	rs1356377759<br>PMID 30131190
FE17004592	HP:0000007-AUTOSOMAL RECESSIVE,<br>HP:0000365-HEARING LOSS,<br>HP:0000407-SENSORINEURAL HEARING LOSS,<br>HP:0011387-DILATED VESTIBULAR AQUEDUCT,<br>HP:0012832-BILATERAL,<br>HP:0031914-FLUCTUATING	*SLC26A4*	NM_000441.1	c.626G>T	p.(Gly209Val)	missense	Heterozygous	4	AR	NO	PMID 9618166<br>PMID 11932316<br>PMID 24224479<br>PMID 26969326
				c.707T>C	p.(Leu236Pro)	missense	Heterozygous	5		NO	rs80338848<br>PMID 9618166<br>PMID 20553101<br>PMID 10861298<br>PMID 26969326<br>PMID 12354788<br>PMID 18310264<br>PMID 20597900<br>PMID 15689455
FE18012228	HP:0000407-SENSORINEURAL HEARING LOSS<br>HP:0000635-BLUE IRIDES<br>HP:0012828-SEVERE<br>HP:0012829-PROFOUND	*EDN3*	NM_207034. 2.	c.476G>T	p.(Cys159Phe)	missense	Homozygous	4	AR	NO	PMID 8630503

Case ID	Phenotype*	Gene symbol	Transcript	Nucleotide change	Protein change	Variant consequence	Variant Zygosity	ACMG Classification(1)	Gene inheritance	Novel at time of analysis (2)	References(3)
FE87188	Congenital sensorineural deafness- progressive.<br>Cochlear implant user.<br>Mild learning disability.	*SLC26A4*	NM_000441.1	c.716T>A	p.(Val239Asp)	missense	Homozygous	4	AR	YES	PMID 12676893<br>PMID 12974744<br>PMID 25394566<br>PMID 30077349<br>PMID 16460646<br>PMID 22116360<br>PMID 27771369
FE19007612	HP:0012265-CILIARY DYSKINESIA	*CCDC40*	NM_017950.3.	c.1416delG	p.(Ile473Phefster2)	frameshift	Homozygous	5	AR	NO	PMID 23255504<br>PMID 23891469<br>PMID 30209139
FE19007613	HP:0000763-SENSORY NEUROPATHY<br>HP:0001156-BRACHYDACTYLY<br>HP:0001251-CEREBELLAR ATAXIA<br>HP:0001260-DYSARTHRIA<br>HP:0001337-TREMOR<br>HP:0007256-PYRAMIDAL SIGNS	*POLR3A*	NM_007055.3.	c.1909+22G>A	p.?	splicing	Heterozygous	4	AR	NO	PMID 27029625<br>PMID 28459997<br>PMID 30564185<br>PMID 30323018<br>PMID 29691679<br>PMID 30847471
				c.3583delG	p.(Asp1195IlefsTer47)	frameshift	Heterozygous	4		NO	rs747683665
FE18013873	HP:0000365-HEARING LOSS<br>HP:0012829-PROFOUND	*CDH23*	NM_022142.5	c.6133G>A	p.(Asp2045Asn)	missense	Homozygous	5	AR	NO	PMID 11090341
FE18018919	HP:0001272-CEREBELLAR ATROPHY<br>HP:0002135-BASAL GANGLIA CALCIFICATION	*TREM2*	NM 018965.3.	c.549delT	p.(Leu184SerfsTer5)	frameshift	Homozygous	3	AD	YES	_________
FE19009211	HP:0000518-CATARACT<br>HP:0000556-RETINAL DYSTROPHY<br>HP:0001083-LENS DISLOCATION	*COL18A1*	NM_130445.2	c.2919dup	p.(Gly974ArgfsTer110)	frameshift	Heterozygous	5	AR	NO	rs768555371
				c.3514_3515delCT	p.(Leu1172ValfsTer72)	frameshift	Heterozygous	5		NO	rs398122391
FE16026130	HP:0000175-CLEFT PALATE<br>HP:0000324-FACIAL ASYMMETRY<br>HP:0000750-SPEECH DELAY<br>HP:0001249-INTELLECTUAL DISABILITY<br>HP:0001999-FACIAL DYSMORPHISM<br>HP:0410030-CLEFT LIP	*CHD7*	NM_017780.3	c.5222G>T	p.(Arg1741Leu)	missense	Heterozygous	3	AD	YES	_________

Case ID	Phenotype*	Gene symbol	Transcript	Nucleotide change	Protein change	Variant consequence	Variant Zygosity	ACMG Classification(1)	Gene inheritance	Novel at time of analysis (2)	References(3)
FE19002968	HP:0000556-RETINAL DYSTROPHY	*SRD5A3*	M_024592.4.	c.57G>A	p.(Trp19Ter)	nonsense	Homozygous	5	AR	NO	rs398124401
FE18030615	HP:0000175-CLEFT PALATE<br>HP:0000365-HEARING LOSS<br>HP:0000823-DELAYED PUBERTY<br>HP:0001263-DEVELOPMENTAL DELAY	*CHD7*	NM_017780.3	c.4353+4A>G	p.?	splicing	Heterozygous	3	AD	YES	__________
FE18032769	HP:0000252-MICROCEPHALY<br>HP:0000280-COARSE FACIES<br>HP:0000996-FACIAL CAPILLARY HEMANGIOMA<br>HP:0001508-FAILURE TO THRIVE<br>HP:0002247-DUODENAL ATRESIA<br>HP:0002509-LIMB HYPERTONIA<br>HP:0009062-INFANTILE AXIAL HYPOTONIA<br>HP:0100704-CORTICAL VISUAL IMPAIRMENT	*GRIN2B*	NM_000834.3	c.2011-1G>A	p.?	splicing	Heterozygous	5	AD	NO	PMID 27605359
FE18018608	Postaxial polydactyly both hands and feet, global developmental delay, echogenic kidneys, possible BBS.	*BBS7*	NM_176824.2.	c.712_715delAGAG	p.(Arg238GlufsTer59)	frameshift	Heterozygous	5	AR	NO	PMID 19402160<br>PMID 26518167<br>PMID 20177705
				c.1967_1968delinsC	p.(Leu656ProfsTer18)	frameshift	Heterozygous	4		NO	
FE19009692	HP:0001181-ADDUCTED THUMBS<br>HP:0001290-HYPOTONIA<br>HP:0001762-TALIPES EQUINOVARUS	*CHST14*	NM_130468.3.	c.652C>A	p.(Arg218Ser)	missense	Heterozygous	3	AD	NO	PMID 25703627
FE14012166	HP:0000365-HEARING IMPAIRMENT<br>HP:0000729-AUTISTIC BEHAVIOR<br>HP:0007483-DEPIGMENTATION/HYPERPIGMENTATION OF SKIN	*CRYM*	NM_001888.3	c.703G>C	p.(Glu235Gln	missense	Heterozygous	3	AD	YES	__________
		FGFR2	NM_000141.4.	c.1032G>A	p.(Ala344Ala)	synonymous	Heterozygous	5	AD	NO	PMID 7987400<br>PMID 8957519<br>PMID 7558045<br>PMID 16158432
FE15003583	Bilateral moderate-to-severe sensorineural hearing loss, cochlear implant user.	*LOXHD1*	NM_144612.6.	c.2295G>A	p.(Trp765Ter)	nonsense	Compound heterozygous	4	AR	YES	__________
				c.6368_6369delCA	p.(Thr2123ArgfsTer30)	frameshift	Compound heterozygous	4		YES	__________
FE18011361	HP:0000252-MICROCEPHALY<br>HP:0002617-ANEURYSM	*ESC02*	NM_001017420.2.	c.1489A>T	p.(Ile497Phe)	missense	Homozygous	3	AR	YES	__________

Case ID	Phenotype*	Gene symbol	Transcript	Nucleotide change	Protein change	Variant consequence	Variant Zygosity	ACMG Classification(1)	Gene inheritance	Novel at time of analysis (2)	References(3)
FE18007064	likely retinal dystrophy	*AHI1*	NM_017651.4	c.2988delA	p.(Val997SerfsTer20)	frameshift	Heterozygous	3	AR	NO	PMID 28041643
FE85265	congenital myopathy	*AMPD1*	NM_000036.2	c.133C>T	p.(Gln45Ter)	nonsense	Homozygous	3	AR	NO	PMID 1631143<br>PMID 21343608
FE15019977	Moderate to severe cerebellar atrophy, brisk lower limb reflexes, clonus, slow speech, learning difficulties.	*KCNA1*	NM_000217.2	c.884G>A	p.(Arg295His)	missense	Heterozygous	3	AD	YES	__________
FE17000781	Bilateral sensorineural hearing loss, bilateral enlarged vestibular acqueduct.	*SLC26A4*	NM_000441.1	c.578C>T	p.(Thr193Ile)	missense	Heterozygous	4	AR	NO	rs111033348<br>PMID 23273637<br>PMID 26752218
FE17005641	Bilateral sensorineural hearing loss<br>and bilateral enlarged vestibular aqueduct.	*SLC26A4*	NM_000441.1	c.349C>T	p.(Leu117Phe)	missense	Heterozygous	4	AR	NO	PMID 26969326<br>PMID 10700480<br>PMID 11932316
FE17002610	HP:0000294 - LOW ANTERIOR HAIRLINE<br>HP:0000582 - UPSLANTED PALPEBRAL FISSURE<br>HP:0000664 - SYNOPHRYS<br>HP:0000885 - BROAD RIBS<br>HP:0001607 - SUBGLOTTIC STENOSIS<br>HP:0002208 - COARSE HAIR<br>HP:0002786 - TRACHEOBRONCHOMALACIA<br>HP:0003016 - METAPHYSEAL WIDENING<br>HP:0003300 - OVOID VERTEBRAL BODIES<br>HP:0004322 - SHORT STATURE<br>HP:0005280 - DEPRESSED NASAL BRIDGE<br>HP:0008430 - ANTERIOR BEAKING OF LUMBAR VERTEBRAE	*GNPTAB*	NM_024312.4	c.3503_3504delTC	p.(Leu1168GlnfsTer5)	frameshift	Heterozygous	4	AR	NO	PMID 16465621<br>PMID 20880125<br>PMID 25788519
FE18017011	Severe Combined Immunodeficiency	*LRBA*	NM_006726.4	c.6584+1delG	p.?	splicing	Heterozygous	4	AR	NO	rs1320366310
FE18032044	Moderate, bilateral sensorineural hearing loss	*OTOA*	NM_144672.3	c.828delT	p.(Ser277ValfsTer3)	frameshift	Heterozygous	4	AR	NO	rs751447996

Case ID	Phenotype*	Gene symbol	Transcript	Nucleotide change	Protein change	Variant consequence	Variant Zygosity	ACMG Classification(1)	Gene inheritance	Novel at time of analysis (2)	References(3)
[illegible]	Moderate, bilateral sensorineural hearing loss										
		MY015A	NM_016239.3	c.5866C>T	p.(Argl956Trp)	MIssense	Heterozygous	3	AR	NO	PMID 26969326
FE14008583	HP:0000407-SENSORINEURAL HEARING IMPAIRMENT<br>HP:0001263-DEVELOPMENTAL DELAY<br>HP:0002066-ATAXIC GAIT<br>HP:0002072-CHOREA<br>HP:0100022-MOVEMENT DISORDER	*CDH23*	NM_022142.5	c.6133G>A	p.(Asp2045Asn)	missense	Heterozygous	5	AR	NO	PMID 11090341
FE14011757	Absent gag reflex, hirsuitism, Mongolian blue spot, dysmorphic features including microcephaly and epicanthic folds, previous flexion contracture of finger	*DHCR7*	NM_001360.2	c.964-1G>C	p.?	splicing	Heterozygous	5	AR	NO	rs138659167
FE15018778	Progressive, profound sensorineural hearing loss	*TMC1*	NM_138691.2	c.1165C>T	p.(Arg389Ter)	nonsense	Heterozygous	4	AR/AD	NO	rs151001642

AR - autosomal recessive, AD: autosomal dominant.
* Phenotypes are described using main clinical description or Human Phenotype Ontology terms entered into the Web Referral System (WRS).
(1): Richards, Sue, et al. "Standards and guidelines for the interpretation of sequence variants: a joint consensus recommendation of the American College of Medical Genetics and Genomics and the Association for Molecular Pathology." Genetics in medicine 17.5 (2015): 405-423.
(2):CES cases analysed between September 2016- December 2019
(3): Reference in published literature (PMID PubMed identifier) and/or reference ID number
